# Loss of Spry1 reduces growth of BRAF^V600^-mutant cutaneous melanoma and improves response to targeted therapy

**DOI:** 10.1038/s41419-020-2585-y

**Published:** 2020-05-22

**Authors:** Barbara Montico, Francesca Colizzi, Giorgio Giurato, Aurora Rizzo, Annamaria Salvati, Lorena Baboci, Dania Benedetti, Eliana Pivetta, Alessia Covre, Michele Dal Bo, Alessandro Weisz, Agostino Steffan, Michele Maio, Luca Sigalotti, Elisabetta Fratta

**Affiliations:** 1 Immunopathology and Cancer Biomarkers, Centro di Riferimento Oncologico di Aviano (CRO), IRCCS, Aviano, Italy; 2 Laboratory of Molecular Medicine and Genomics, Department of Medicine, Surgery and Dentistry ‘Scuola Medica Salernitana’, University of Salerno, Baronissi, SA Italy; 3 Genomix4Life Srl, University of Salerno, Baronissi, SA Italy; 4 Experimental and Clinical Pharmacology Unit, Centro di Riferimento Oncologico di Aviano (CRO), IRCCS, Aviano, Italy; 5 Clinical and Experimental Onco-Hematology Unit, Centro di Riferimento Oncologico di Aviano (CRO), IRCCS, Aviano, Italy; 6 Molecular Oncology, Centro di Riferimento Oncologico di Aviano (CRO), IRCCS, Aviano, Italy; 7 Center for Immuno-Oncology, Medical Oncology and Immunotherapy, Department of Oncology, University Hospital of Siena, Siena, Italy; 8 Fondazione Toscana Life Sciences, Siena, Italy; 9 NIBIT Foundation Onlus, Siena, Italy; 10 Oncogenetics and Functional Oncogenomics Unit, Centro di Riferimento Oncologico di Aviano (CRO), IRCCS, Aviano, Italy

**Keywords:** Melanoma, Preclinical research

## Abstract

Mitogen-activated protein kinase (MAPK) pathway activation is a central step in BRAF^V600^-mutant cutaneous melanoma (CM) pathogenesis. In the last years, Spry1 has been frequently described as an upstream regulator of MAPK signaling pathway. However, its specific role in BRAF^V600^-mutant CM is still poorly defined. Here, we report that Spry1 knockdown (Spry1^KO^) in three BRAF^V600^-mutant CM cell lines markedly induced cell cycle arrest and apoptosis, repressed cell proliferation in vitro, and impaired tumor growth in vivo. Furthermore, our findings indicated that Spry1^KO^ reduced the expression of several markers of epithelial–mesenchymal transition, such as MMP-2 both in vitro and in vivo. These effects were associated with a sustained and deleterious phosphorylation of ERK1/2. In addition, p38 activation along with an increase in basal ROS levels were found in Spry1^KO^ clones compared to parental CM cell lines, suggesting that BRAF^V600^-mutant CM may restrain the activity of Spry1 to avoid oncogenic stress and to enable tumor growth. Consistent with this hypothesis, treatment with the BRAF inhibitor (BRAFi) vemurafenib down-regulated Spry1 levels in parental CM cell lines, indicating that Spry1 expression is sustained by the MAPK/ERK signaling pathway in a positive feedback loop that safeguards cells from the potentially toxic effects of ERK1/2 hyperactivation. Disruption of this feedback loop rendered Spry1^KO^ cells more susceptible to apoptosis and markedly improved response to BRAFi both in vitro and in vivo, as a consequence of the detrimental effect of ERK1/2 hyperactivation observed upon Spry1 abrogation. Therefore, targeting Spry1 might offer a treatment strategy for BRAF^V600^-mutant CM by inducing the toxic effects of ERK-mediated signaling.

## Introduction

Cutaneous melanoma (CM) is a very aggressive malignancy that still represents the deadliest form of skin cancer^1^. About 50% of CM harbors the activating BRAF^V600^ mutation which exerts most of the oncogenic effects through the mitogen-activated protein kinase (MAPK) signaling pathway^2^. Although targeted therapy directed against BRAF has recently shown clinical effectiveness, CM patients invariably develop an early drug resistance^3^. Accordingly, a better understanding of molecular mechanisms involved in MAPK regulation would likely facilitate the development of more effective therapeutic strategies for BRAF^V600^-mutant CM patients.

In the last years, a number of MAPK modulators have been identified, including Sprouty (Spry) proteins. The Spry family members are products of four genes located on different chromosomes^4^, and differ in their tissue distribution, activity, and interaction partners^5^. All four Spry proteins share a cysteine-rich domain that is likely to confer an inhibitory activity of Ras–MAPK signaling^6^, whereas differences in the N-terminal region may dictate their functional divergence^4^. Spry proteins are not able to fully complement each other^7^, and their biological function is likely to be dependent on tissue and cell type context^8^. Although Spry proteins have been found to interact with several Ras–MAPK pathway components^9^, the way through which they modulate Ras–MAPK signaling has not been fully elucidated yet. Spry1 was initially identified as an inhibitor of the Ras–MAPK pathway in *Drosophila melanogaster*^10,11^. Surprisingly, subsequent studies demonstrated that Spry proteins not only inhibited Ras–MAPK pathway^12–16^ but also enhanced the activation of this pathway in a context-specific manner^17,18^. In addition, Spry genes themselves appear to be transcriptionally controlled by Ras–MAPK signaling cascade. In fact, down-regulation of Spry proteins has been observed upon MEK1/2 inhibition, thus confirming their expression to critically depend on ERK1/2 activity^19–21^. Spry1 function in cancer has proven elusive, and it is likely dependent on tumor type, and genetic context. In fact, although a reduced expression of Spry1 was found in sarcoma^22^, liver^23^, ovarian^24^, and prostate cancers^25^, accumulating evidence indicate that up-regulation of Spry proteins, including Spry1, can promote the growth of various tumors harboring Raf or Ras mutations where they act as enhancers rather than as inhibitors of the Ras–MAPK pathway^26–29^. In this context, the involvement of Spry1 in CM has been poorly investigated so far^30^. In this study we explored the role of Spry1 in BRAF^V600^-mutant CM. Through in vitro and in vivo assays, we demonstrated that Spry1 knockout (Spry1^KO^) could efficiently reduce the viability of BRAF^V600^-mutant CM, and improve response to targeted therapy. These results indicate that Spry1 can contribute in at least a subset of CM to promote the malignant phenotype, and may thus represent a novel therapeutic target in BRAF^V600^-mutant CM.

## Results

### Spry1 is highly expressed in BRAF^V600^-mutant CM tissues and cell lines

**Fig. 1:**
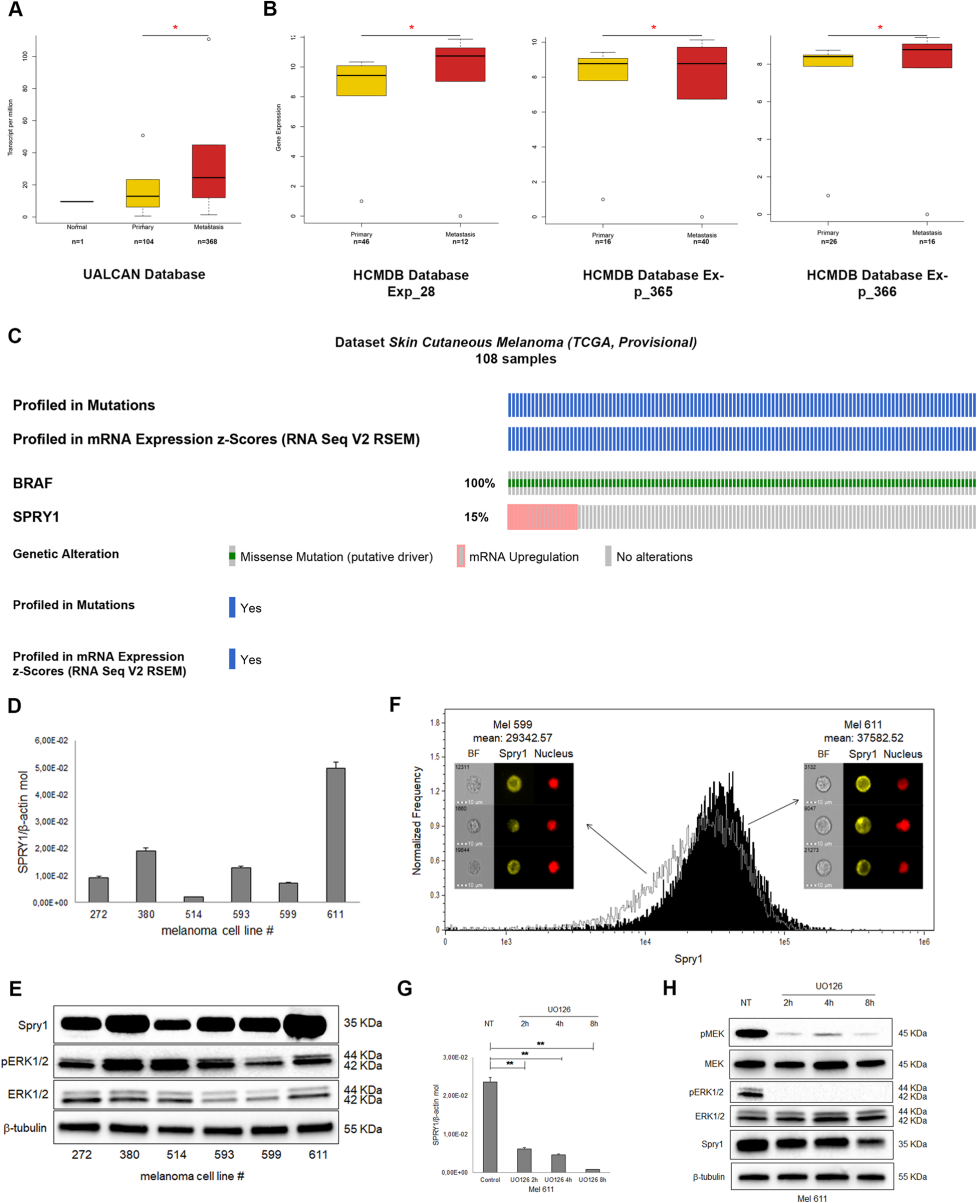
Spry1 expression in CM and in BRAF^V600^-mutant CM. **a**, **b** Box plots showing the expression of Spry1 gene in normal tissues, and in primary and metastatic CM considering data taken from UALCAN Database (**a**), and in primary and metastatic CM for selected experiments taken from HCMDB Database (**b**). Statistically significant differences were indicated: *p < 0.01. **c** OncoPrint showing Spry1 gene expression in BRAF^V600^-mutant CM samples taken from TCGA. **d** mRNA expression of Spry1 was analyzed by qRT-PCR assay in six BRAF^V600^-mutant CM cell lines, and normalized to β-actin. Each bar represents *n* = 2 biological replicates, three technical replicates each; mean ± standard deviation (SD). **e** The levels of Spry1, phospho-ERK1/2 (pERK1/2), and total ERK1/2 protein were determined by western blot in six BRAF^V600^-mutant CM cell lines. β-Tubulin was used as a loading control. Western blot images are representative of three independent experiments. **f** Cytoplasmic localization of Spry1 in Mel 599 and Mel 611 CM cell lines. 3 × 10^5^ cells were acquired and analyzed with the ImageStreamX instrument. **g**, **h** Mel 611 cells were treated or not (NT) with the MEKi U0126 (50 μmol/L) for 2, 4, and 8 h, and then the expression of SPRY1 was determined by qRT-PCR (**g**) and western blot analyses (**h**). Each bar represents *n* = 3 biological replicates, 3 technical replicates each; means ± SD. Statistically significant differences were indicated: **p < 0.01. A representative blot of three independent experiments with similar results were shown.

The expression of Spry1 in human CM was initially explored using publicly available cancer gene expression profiling and transcriptome sequencing data. The Cancer Genome Atlas (TCGA) data for Spry1 were firstly obtained using the UALCAN web portal for gene expression analyses (http://ualcan.path.uab.edu/)^31^, which showed the messenger RNA (mRNA) expression of Spry1 was significantly elevated in metastatic CM respect to primary tumors (*p* value<0.01) (Fig. 1a). To further confirm these data the mRNA expression of Spry1 was analyzed by using the Human Cancer Metastasis Database (HCMDB) (http://hcmdb.i-sanger.com/index)^32^, and the results of GSE15605 (Exp_00028) and GSE7553 (Exp_00365 and Exp_00366) datasets demonstrated that the mRNA levels of Spry1 were significantly up-regulated in metastatic CM compared with primary lesions (*p* value <0.01) (Fig. 1b). Given Spry2 was found to promote the growth of tumors harboring BRAF mutations^27^, we analyzed Spry1 expression in BRAF^V600^-mutant CM by using cBioPortal (http://www.cbioportal.org/)^33^, and overexpression of Spry1 mRNA was observed in 15% of these tumor types (Fig. 1c).

The expression levels of Spry1 mRNA was then examined in a panel of CM cell lines carrying the BRAF^V600^ mutation (Supplementary Table 1) and wild type for NRAS. As shown in Fig. 1d, Spry1 mRNA was detected in all CM cell lines tested, with Mel 611 cells expressing the highest level. Interestingly, Spry1 mRNA expression well correlated with protein levels in all BRAF^V600^-mutant CM cell lines (Fig. 1e), and was predominantly localized in the cytosol (Fig. 1f).

Since it has been reported that Spry proteins can be induced by the MAPK/ERK pathway^34^, we analyzed ERK1/2 phosphorylation and, as shown in Fig. 1e, ERK1/2 was activated in all BRAF^V600^-mutant CM cell lines expressing Spry1. To evaluate whether a causal link connects MAPK/ERK activity and Spry1 expression, Mel 611 cell line was treated with the MEK inhibitor (MEKi) UO126 for 2, 4, and 8 h. Treatment abrogated MEK–ERK phosphorylation, and both mRNA and protein levels of Spry1 were reduced in a time-dependent manner (Fig. 1g, h). These results strongly suggest that Spry1 expression is transcriptionally controlled by MAPK/ERK signaling.

### Successful knockout of Spry1 in BRAF^V600^-mutant CM cell lines using CRISPR/Cas9 strategy

**Fig. 2:**
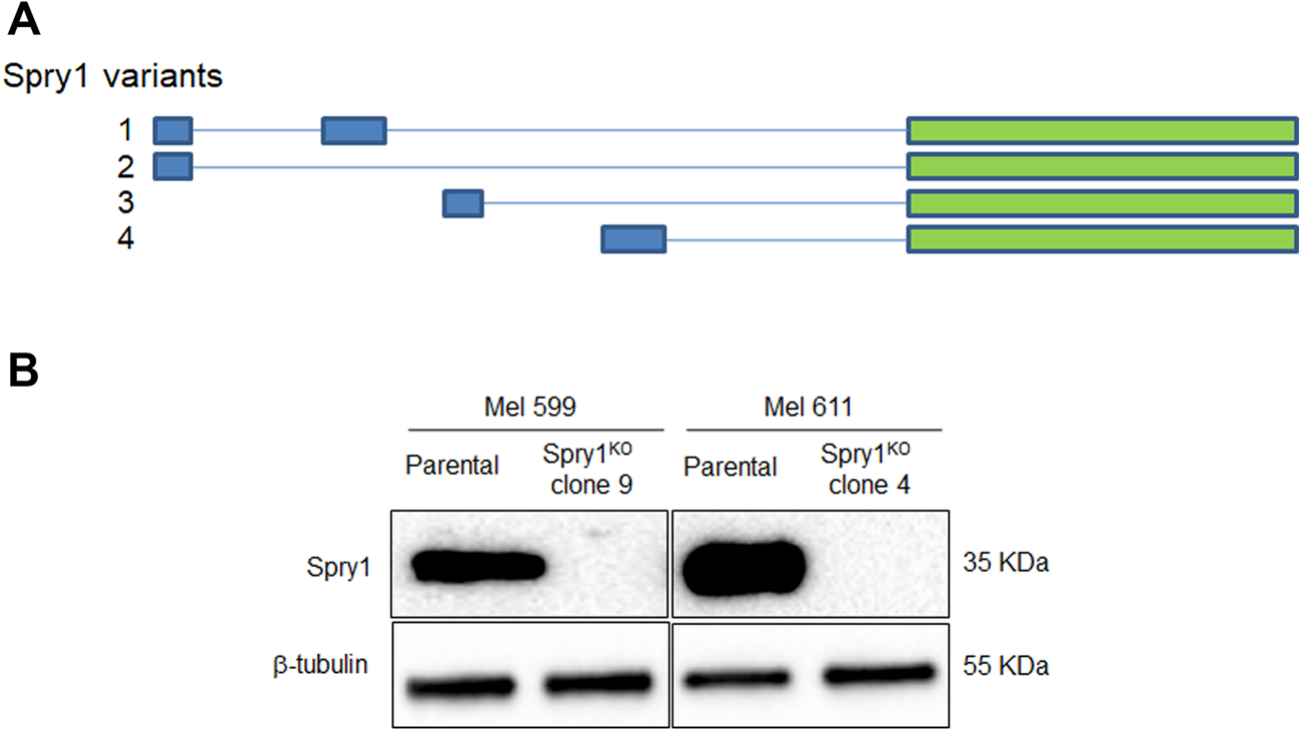
Genomic editing by CRISPR/Cas9 in BRAF^V600^-mutant CM cell lines. **a** Schematic illustration of structure of the four Spry1 transcripts. Green boxes indicate the common exon encoding Spry1 protein. **b** Spry1 expression was evaluated by western blot analysis in parental BRAF^V600^-mutant CM cell lines and respective Spry1^KO^ clones. β-Tubulin was used as a loading control.

Spry1 exists in four transcript variants, all of which contain the same coding exon, and encode the same protein (Fig. 2a). To explore the biological functions of Spry1 in BRAF^V600^-mutant CM, stable Spry1^KO^ gene was achieved using the CRISPR/CAS9 strategy in the two BRAFi sensitive CM cell lines Mel 611, which expressed the highest levels of Spry1, and Mel 599 that, besides BRAF^V600^ mutation, carried other two point mutations: a homozygous mutation in the splice acceptor site of TP53 intron 9 that predicts to alter splicing (Supplementary Fig. S1) and a heterozygous nucleotide substitution (121A>G) into the exon 3 of the β-catenin gene neighboring an important phosphorylation site (Thr-41) that results in β-catenin stabilization^35^ (Supplementary Fig. S2).

Through a series of screenings, clones with complete loss of Spry1 protein expression were successfully established in both Mel 599 (Mel 599 Spry1^KO^ clone 9) and Mel 611 cell lines (Mel 611 Spry1^KO^ clone 4), with gRNA#1 working best for the suppression (Fig. 2b).

### Spry1^KO^ decreases cell proliferation, induces apoptosis, and promotes MAPK pathway hyperactivation in BRAF^V600^-mutant CM

**Fig. 3:**
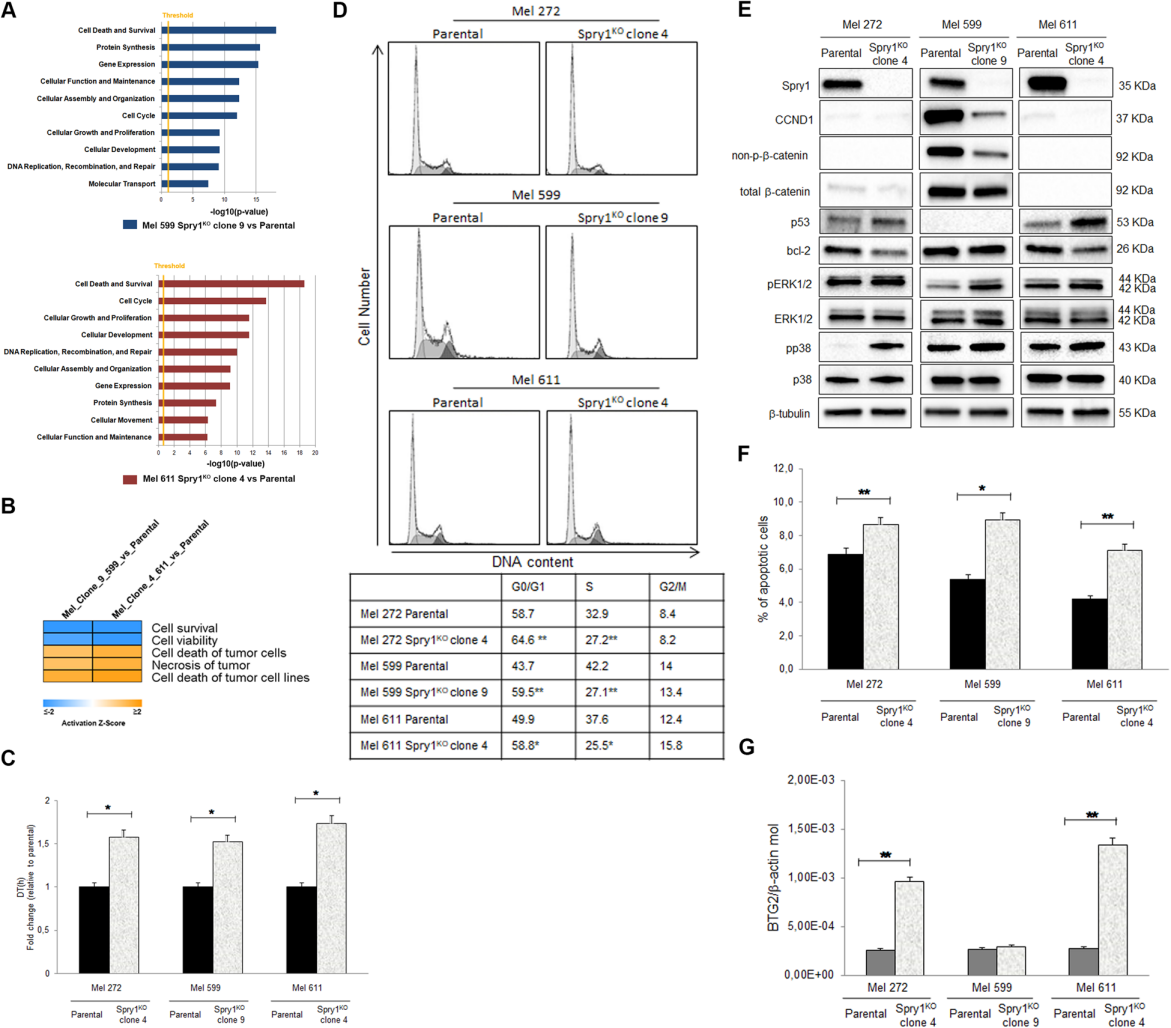
Spry1 knockout inhibited cell proliferation and promoted apoptosis of BRAF^V600^-mutant CM cells. **a** Histograms showing the top functional categories where the common differentially expressed genes in Mel 599 and Mel 611 Spry1^KO^ clones are involved. **b** Heatmap showing the activation (or inhibition) *Z*-score for specific subcategories of “Cell Death and Survival” obtained using IPA, and considering the differentially expressed genes in Mel 599 and Mel 611 Spry1^KO^ clones with respect to their parental cells. **c** Cell proliferation analysis over an incubation time of 96 h by the xCELLigence real-time cell analyzer in parental CM cell lines and Spry1^KO^ clones. Doubling time (DT) values of Spry1^KO^ clones with respect to parental cells are shown. All the experiments were performed in triplicate and are presented as fold average ± standard deviation. Statistically significant differences were indicated: *p < 0.05. **d** Parental CM cell lines and respective Spry1^KO^ clones were cultured for 48 h, and then flow cytometry was used to assess the cell cycle distribution. Percentage (%) of cells in each phase of the cycle is shown. Data are the representative of at least three independent experiments. Statistically significant differences were indicated: *p < 0.05, **p < 0.01. **e** Western blot analysis of Spry1, cyclin D1 (CCND1), non-phosphorylated serine (Ser)-33/Ser-37/Thr-41 β-catenin (non-p-β-catenin), total β-catenin, p53, bcl2, phospho-ERK1/2 (pERK1/2), ERK1/2, phospho-p38 (pp38), and p38 in parental BRAF^V600^-mutant CM cell lines and respective Spry1^KO^ clones. β-Tubulin was used as a loading control. Western blot images are representative of three independent experiments. **f** Percentage of apoptotic cells. Shown are means of at least three independent experiments ±SD. Statistically significant differences were indicated: *p < 0.05, **p < 0.01. **g** qRT-PCR analyses of BTG2 expression in CM cell lines and their relative Spry1^KO^ clones. Total RNA was extracted for reverse transcription, analyzed by qRT-PCR, and normalized to β-actin. Each bar represents *n* = 2 biological replicates, 3 technical replicates each; means ± SD. Statistically significant differences were indicated: **p < 0.01.

To gain insight into the changes in gene expression associated with Spry1^KO^, we investigated differences in the transcriptional landscape between Spry1^KO^ clones and parental cells. Ingenuity Pathway Analysis (IPA) of genes differentially expressed between Spry1^KO^ clones and parental cells identified cell death and survival as the most significant molecular and cellular function affected by Spry1 silencing (Fig. 3a). In particular, results from IPA analysis indicated that “Cell Survival and Viability” functions resulted inhibited, whereas “Cell Death of Tumor Cells and Necrosis” functions were predicted to be activated in Spry1^KO^ clones with respect to their controls (Fig. 3b). Therefore, we assessed whether Spry1^KO^ could inhibit BRAF^V600^-mutant CM cell growth in both Mel 599 and Mel 611 Spry1^KO^ clones, and in a Spry1^KO^ clone generated from the more aggressive Mel 272 cell line (Supplementary Figure S3) that expressed high levels of Spry1 protein, carried the V600K mutation, which is a rare two-nucleotides substitution (Supplementary Table 1, Supplementary Fig. S4), and was less sensitive to vemurafenib treatment. We conducted cell proliferation analysis by a xCELLigence real-time cell analyzer and confirmed that Spry1 depletion effectively reduced the cell proliferation rate in all Spry^KO^ clones compared to parental cells (Fig. 3c). Consistently, cell cycle analyses revealed a significant accumulation of Spry1^KO^ cells in the G1 phase with a concomitant decrease of those in the S phase (Fig. 3d). Since cyclin D1 (CCND1) is essential for cell cycle progression in G1/S, its expression was evaluated by western blot analysis. Mel 611 and Mel 272 parental cells expressed barely detectable levels of CCND1, and thus, no further reduction could be observed following Spry1^KO^. In contrast, Spry1^KO^ resulted in a dramatic reduction of CCND1 protein in the CCND1 highly positive Mel 599 cells (Fig. 3e). Aberrant β-catenin activation has been shown to transactivate CCND1 (ref. ^36^); therefore, we sought to verify whether Spry1^KO^ affected the β-catenin pathway. Results demonstrated that Mel 599 Spry1^KO^ clone 9 exhibited a significant decrease of non-phosphorylated serine (Ser)-33/Ser-37/Thr-41 β-catenin (Fig. 3e), indicating that reduced CCND1 expression following Spry1^KO^ was likely mediated by β-catenin inhibition. Next, we evaluated the apoptotic effects of Spry1 depletion and, as shown in Fig. 3f, a slight increase in the proportion of apoptotic cells was detected in Spry1^KO^ clones. A reduced expression of bcl2 protein along with a substantial raise in total levels of p53 protein was observed in Mel 272 and Mel 611 Spry1^KO^ clones, but not in the TP53-mutated Mel 599 Spry1^KO^ clone 9 (Fig. 3e). Furthermore, the mRNA expression level of BTG2, a p53-transcriptional target^37^, was also up-regulated in Mel 272 and Mel 611 Spry1^KO^ clones (Fig. 3g). Collectively, our data indicated that Spry1^KO^ altered cell cycle dynamics and promoted apoptosis in BRAF^V600^-mutant CM cell lines even in the presence of additional mutations, through both p53-dependent and -independent mechanisms.

In several different cellular contexts, loss of Spry1 gene function results in hyperactive MAPK/ERK signaling^38^. Consistent with these published data, ERK1/2 was more activated in Spry1^KO^ clones (Fig. 3e, Supplementary Fig. S5). Although ERK1/2 activation has generally been associated with cell survival and proliferation^39^, the above results demonstrated that Spry1 silencing associates with reduced proliferation of BRAF^V600^-mutant CM cells. Interestingly, phosphorylation of the p38 stress MAPK was also significantly increased in Spry1^KO^ clones compared to parental cells (Fig. 3e, Supplementary Fig. S5). Thus, the reduced proliferation upon Spry1 depletion might be due to oncogenic stress triggered by hyperactivation of MAPK signaling pathways in BRAF^V600^-mutant CM cells.

### Spry1^KO^ impairs the migration ability of BRAF^V600^-mutant CM and affects the expression of epithelial–mesenchymal transition-related markers

**Fig. 4:**
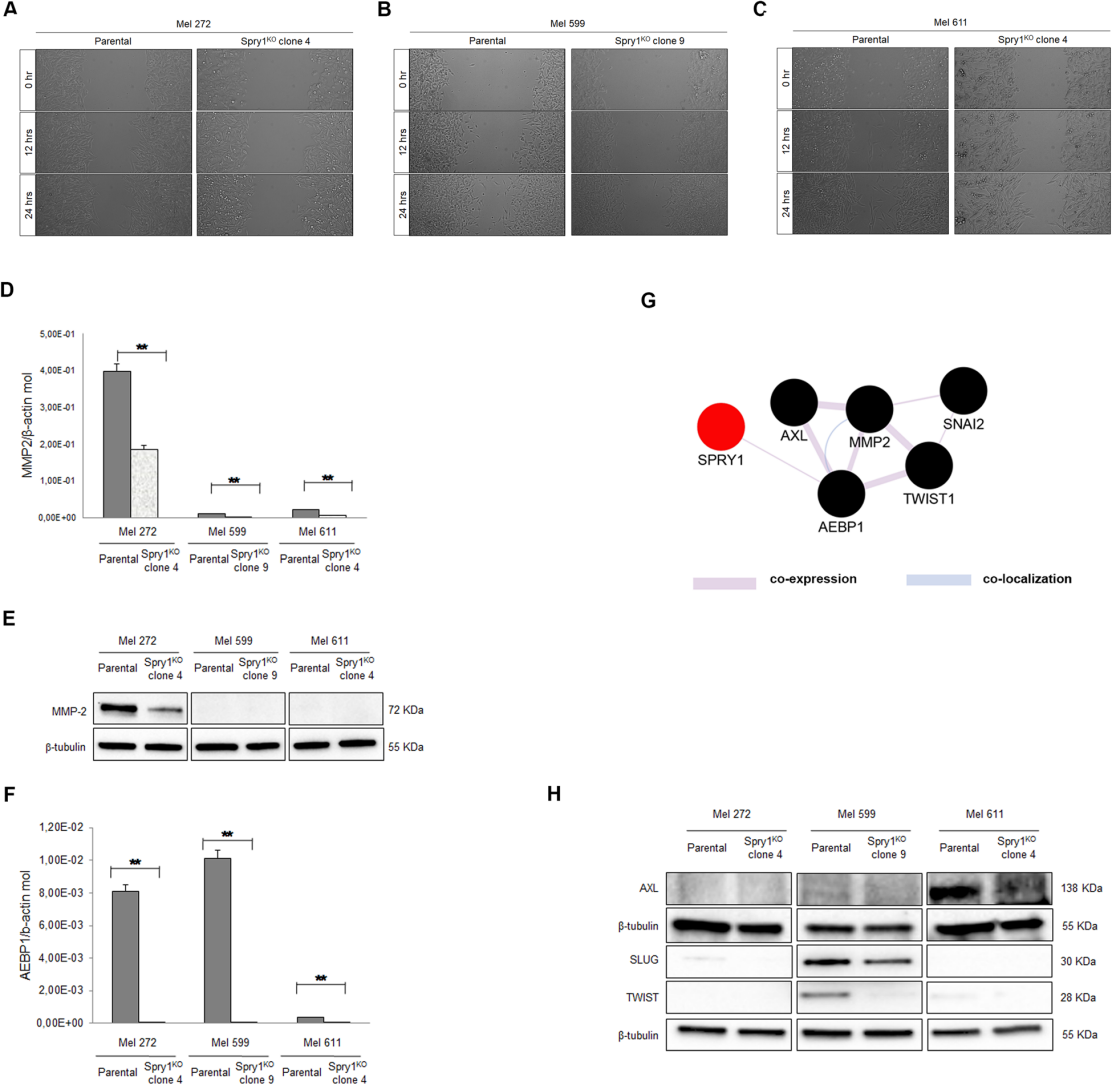
Spry1 knockout reduced cell migration of BRAF^V600^-mutant CM cells. **a**–**c** Representative images of wound healing migration assays for Mel 272 (**a**), Mel 599 (**b**), and Mel 611 (**c**) parental cells and their relative Spry1^KO^ clones. **d** qRT-PCR analysis of MMP-2 expression in CM cell lines and their relative Spry1^KO^ clones. Total RNA was extracted for reverse transcription, analyzed by qRT-PCR, and normalized to β-actin. Each bar represents *n* = 2 biological replicates, 3 technical replicates each; means ± SD. Statistically significant differences were indicated: **p < 0.01. **e** Western blot detection of MMP-2 protein in SPRY1^KO^ clones and their relative parental cells. β-Tubulin was used as a loading control. **f** qRT-PCR analysis of AEBP1 expression in CM cell lines and their relative Spry1^KO^ clones. Total RNA was extracted for reverse transcription, analyzed by qRT-PCR, and normalized to β-actin. Each bar represents n = 2 biological replicates, 3 technical replicates each; means ± SD. Statistically significant differences were indicated: **p < 0.01. **g** The co-expression network of Spry1, AEBP1, AXL, MMP-2, SLUG (SNAI2), and TWIST1 based on GeneMANIA. Co-expression: two genes are linked if their expression levels are similar across conditions in a gene expression study. **h** Western blot detection of AXL, SLUG, and TWIST proteins in SPRY1^KO^ clones and their relative parental cells. β-Tubulin was used as a loading control.

To determine whether Spry1^KO^ had also an effect on the migration of BRAF^V600^-mutant CM cells, wound healing assays were performed. As shown in Fig. 4a–c, Spry1^KO^ impaired the ability of clones to close the wound, whereas parental cell lines closed the wound almost completely. Interestingly, to compare genes that were commonly modulated in Mel 599 and Mel 611 Spry1^KO^ clones indicated (Supplementary Table 2) that the expression of the matrix metalloproteinase 2 (MMP-2) was reduced following Spry1^KO^. Since MMP-2 contributes to migration and invasion of BRAF^V600^-mutant CM^40^, MMP-2 mRNA and protein levels were evaluated. Although quantitative reverse transcriptase PCR (qRT-PCR) showed detectable MMP-2 mRNA in all parental cell lines and its reduction in all Spry1^KO^ clones (Fig. 4d), MMP-2 protein was found strongly expressed only in the highly aggressive Mel 272 cell line, but significantly reduced in the corresponding Spry1^KO^ clone (Fig. 4e). Besides MMP-2, also the adipocyte enhancer-binding protein 1 (AEBP1) was commonly down-regulated in Spry1^KO^ clones (Supplementary Table 2). AEBP1 has been recently reported to be up-regulated in various cancers^41–44^, including vemurafenib-resistant CM cells^45^. In addition, AEBP1 silencing has been recently associated to reduced migration, metastasis, and epithelial–mesenchymal transition (EMT) of gastric cancer cells^46^. AEBP1 was then verified through qRT-PCR and, as shown in Fig. 4f, its constitutive expression was significantly down-regulated in Spry1^KO^ clones. Notably, we found that AEBP1 is co-expressed with Spry1, but also with several EMT-markers (i.e., AXL, TWIST, SLUG), and MMP-2 as well (Fig. 4g). Therefore, the EMT-markers AXL, TWIST, and SLUG were evaluated and, although they were heterogeneously expressed among Mel 272, Mel 599, and Mel 611 CM cell lines, their protein expression profiles were significantly decreased in the respective Spry1^KO^ clones (Fig. 4h). These data indicated that Spry1 abrogation altered the expression of genes involved in EMT in BRAF^V600^-mutant CM.

### Tumorigenicity of BRAF^V600^-mutant CM cells is impaired by Spry1^KO^ in vivo

**Fig. 5:**
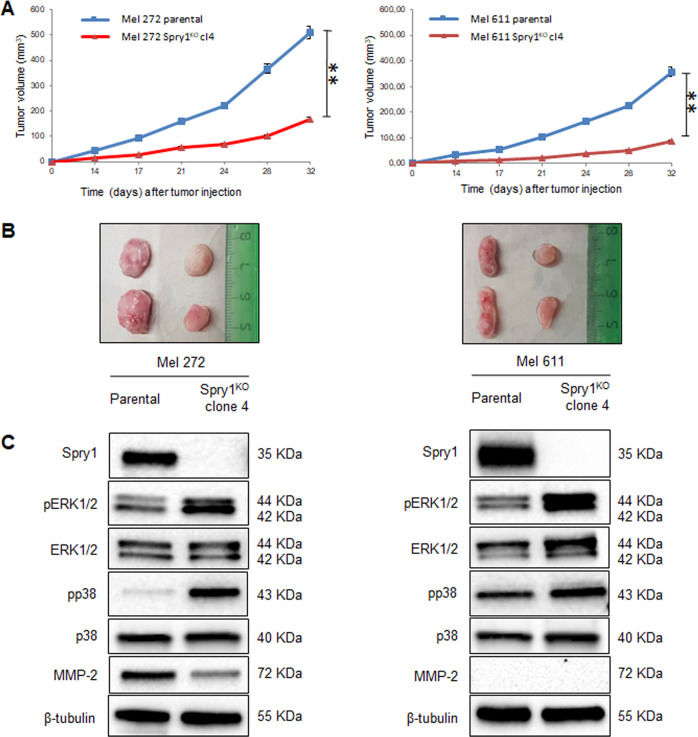
Mel 272 and Mel 611 parental and Spry1^KO^ cells were injected subcutaneously into the right flank of nude mice (*n* = 4 each group). **a** Changes in the mean of tumor volumes have been shown in the tumor growth curve. The data reflect representative of two independent experiments. Data are presented as means ± SD. Statistically significant differences were indicated: **p < 0.01. Approximately 1 month after implantation, tumors were excised and processed for western blots. **b** Two representative tumors from each experimental group has been presented to reflect tumor sizes. **c** Western blot analyses of Spry1, phospho-ERK1/2 (pERK1/2), ERK1/2, phospho-p38 (pp38), p38, and MMP-2 in tumor tissues. β-Tubulin was used as a loading control. Western blot images are representative of three different tumor tissues from each group.

To investigate whether Spry1 was important for the tumorigenicity of BRAF^V600^-mutant CM cells in vivo, Spry1^KO^ clones and their parental cells were subcutaneously inoculated into the right flank of six-week-old female athymic nude mice. Tumor growth was monitored at least twice per week, and documented once tumor growth became visible. Mel 272 and Mel 611 Spry1^KO^ clones gave rise to tumors in all mice, whereas Mel 599 Spry1^KO^ clone 9 did not. As shown in Fig. 5a, tumors arising from Mel 272 and Mel 611 Spry1^KO^ cells were significantly smaller than those from parental cells throughout the course of the experiment. Some representative images reflecting tumor size from experimental groups at the end of the study are also presented (Fig. 5b). Tumors were excised one month after injection and, consistent with our in vitro findings, protein analyses of tumor tissues revealed that Spry1^KO^ associated to decreased MMP-2 protein expression, and to enhanced MAPK/ERK and p38/MAPK phosphorylation (Fig. 5c). These data demonstrate that Spry1^KO^ reduces the ability of BRAF^V600^-mutant CM cells to form tumors in xenotransplant assay.

### Spry1^KO^ evokes cellular oxidative stress in BRAF^V600^-mutant CM cells

**Fig. 6:**
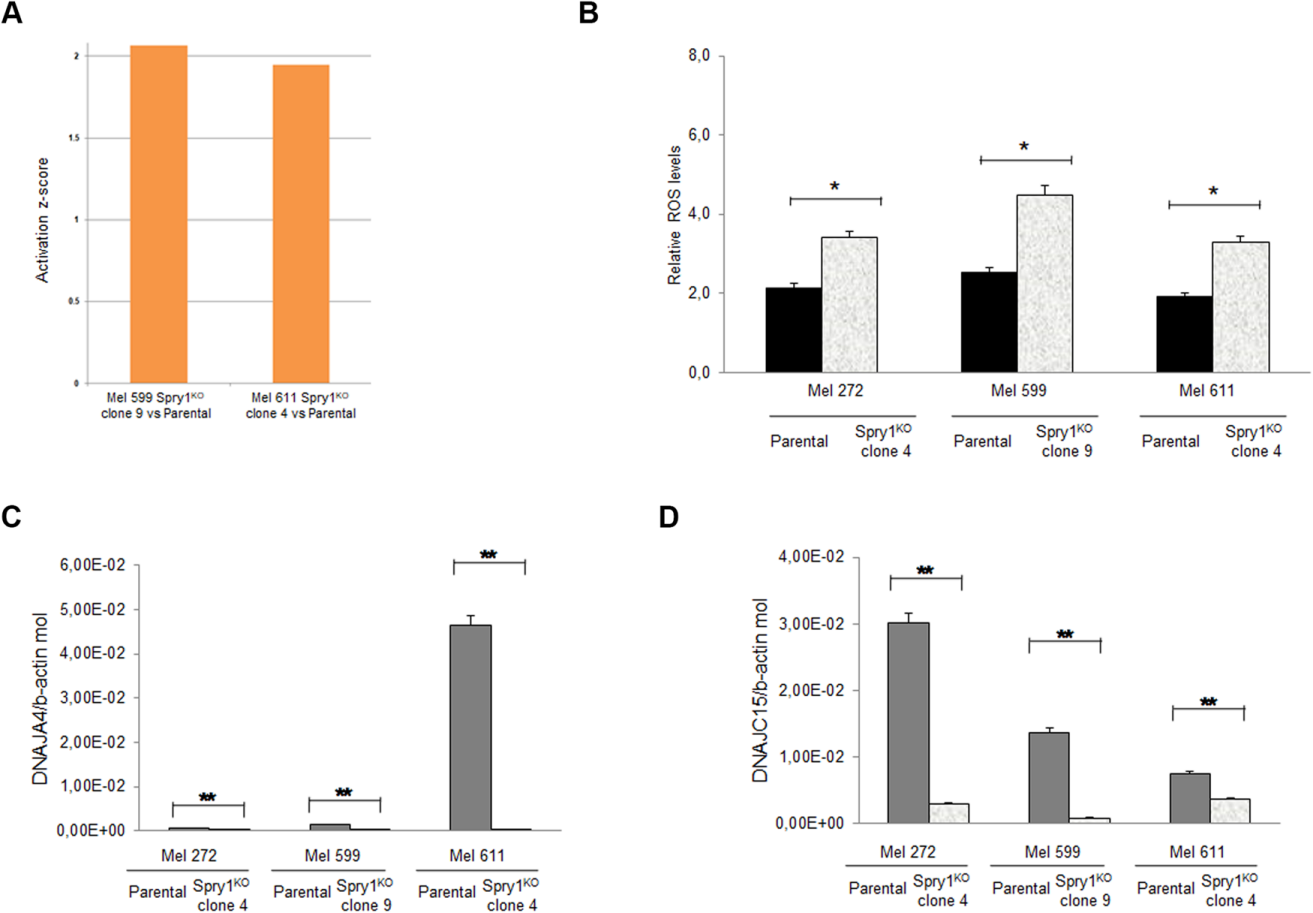
Induction of oxidative stress in BRAF^V600E^-mutant CM cells following Spry1^KO^. **a** Histogram showing the activation *Z*-score of “Production of Nitric Oxide and Reactive Oxygen Species in Macrophages” computed using IPA, and considering the differentially expressed genes in Mel 599 and Mel 611 Spry1^KO^ clones with respect to their parental cells. **b** ROS levels were examined using flow cytometry in parental BRAF^V600^-mutant CM cell lines and respective Spry1^KO^ clones. Images are representative of three independent experiments. Data are mean ± SD of at least three independent experiments. Statistically significant differences were indicated: *p < 0.05. **c**, **d** qRT-PCR analyses of DNAJA4 (**c**) and DNAJC15 (**d**) expression in CM cell lines and their relative Spry1^KO^ clones. Total RNA was extracted for reverse transcription, analyzed by qRT-PCR, and normalized to β-actin. Each bar represents *n* = 2 biological replicates, 3 technical replicates each; means ± SD. Statistically significant differences were indicated: **p < 0.01.

Patel et al.^47^ have recently described an increase in the level of reactive oxygen species (ROS) production following Spry2 silencing. In line with this notion, querying of RNA-seq data predicted the “Production of Nitric Oxide and ROS in Macrophages” function to be activated in Mel 599 and Mel 611 Spry1^KO^ clones compared to parental cells (Fig. 6a). Therefore, we examined whether Spry1 depletion affected the level of intracellular ROS production. Flow cytometry experiments showed that basal ROS levels were effectively augmented in Spry1^KO^ clones compared to parental cells, with Mel 599 Spry1^KO^ clone 9 showing the maximal increase (Fig. 6b). We also observed that the expression of some anti-oxidative response genes, e.g. the DnaJ family members, was commonly down-regulated in Spry1^KO^ clones (Fig. 6c, d).

### Spry1^KO^ increases sensitivity of BRAF^V600^-mutant CM cells to targeted therapy in vitro and in vivo

**Fig. 7:**
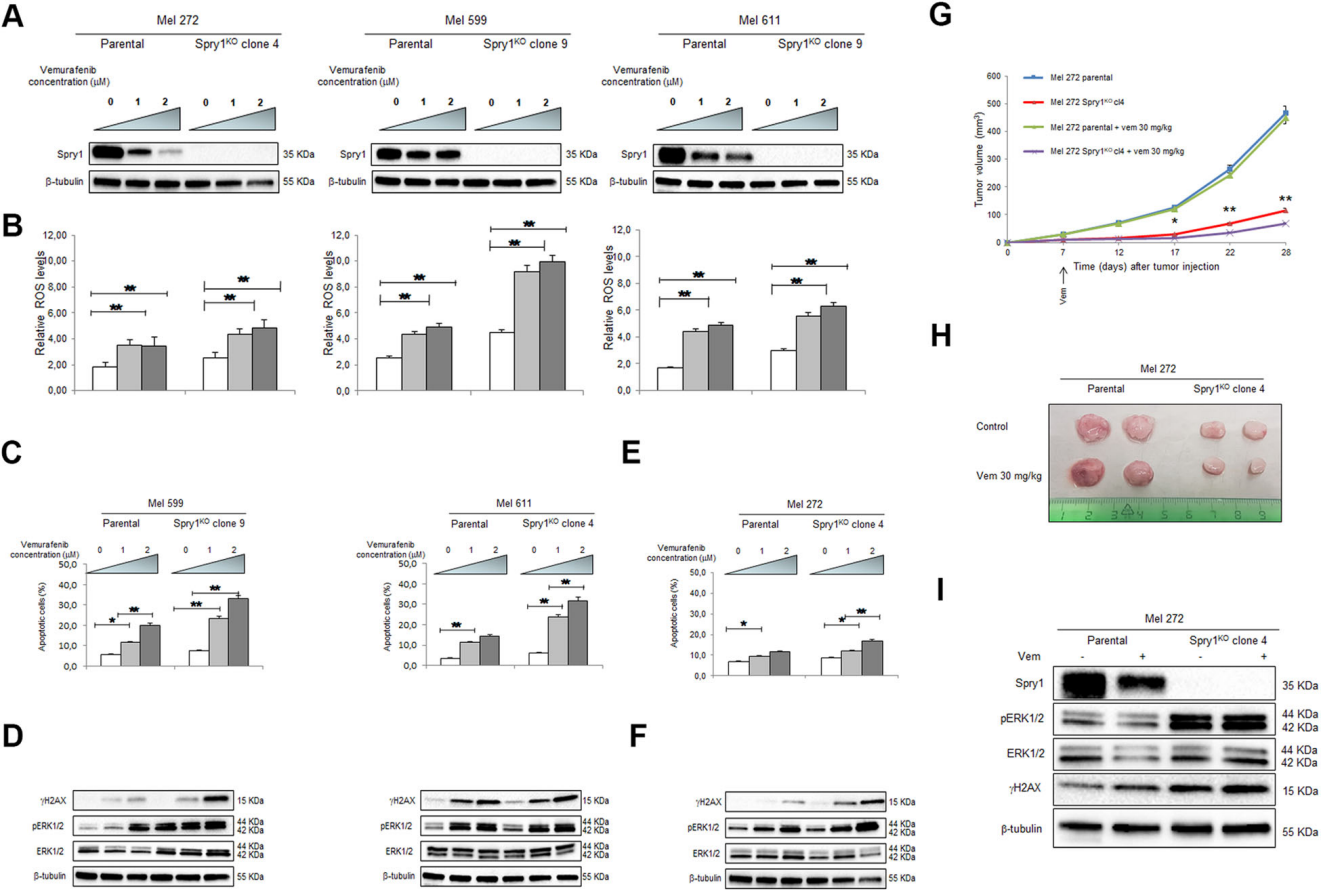
Effects of vemurafenib treatment on parental CM cell lines and respective Spry1^KO^ clones. **a**, **b** Cells were treated with increasing doses of vemurafenib (0–2 µM) for 3 days, and then analyzed for Spry1 protein expression (**a**) and ROS detection (**b**). Western blot images are representative of three independent experiments (**a**). Data are mean ± SD of three independent experiments. Statistical significant differences were indicated: **p < 0.01 (**b**). **c**, **d** Mel 599 and Mel 611 CM cell lines and their respective clones were treated with vemurafenib at the indicated concentrations for 3 days. **c** Apoptosis was measured by annexin V (Ann-V) and propidium iodide (PI) staining through flow cytometry. Shown are means of three independent experiments ± SD. Statistically significant differences were indicated: *p < 0.05, **p < 0.01. **d** Expression of γH2AX, phospho-ERK1/2 (pERK1/2), and ERK1/2 was determined by western blot. β-Tubulin was used as a loading control. A representative blot of three independent experiments with similar results were shown. **e**, **f** Mel 272 cell line and Spry1^KO^ clone 4 were treated with vemurafenib at the indicated concentrations for 5 days. **e** Apoptosis was measured by annexin V (Ann-V) and propidium iodide (PI) staining through flow cytometry. Shown are means of three independent experiments ± SD. Statistically significant differences were indicated: *p < 0.05, **p < 0.01. **f** Expression of γH2AX, phospho-ERK1/2, and ERK1/2 was determined by western blot. β-Tubulin was used as a loading control. Western blot images are representative of three independent experiments. **g–i** Mel 272 parental and Spry1^KO^ cells were injected subcutaneously into the flank of nude mice. When palpable tumors appeared (approximately in 1 week), animals (*n* = 4–6 each group) were injected intraperitoneally with vemurafenib (30 mg/kg/day); animals in the control group were treated with vehicle. Treatments were continued for 21 days. **g** Changes in the mean of tumor volumes have been shown in the tumor growth curve. Data are presented as means ± SD. Statistically significant differences were indicated: *p < 0.05, **p < 0.01. Tumors were harvested at the end of the experiment, and processed for western blots. **h** Two representative tumors from each experimental group has been presented to reflect tumor sizes. **i** Western blot analyses of Spry1, phospho-ERK1/2 (pERK1/2), ERK1/2, and γH2AX **i**n tumor tissues. β-Tubulin was used as a loading control. Western blot images are representative of three different tumor tissues from each group.

Vemurafenib was reported to significantly down-regulate Spry2 and Spry4 expression in BRAF^V600^-mutant CM^48^, but also to stimulate some ROS-therapeutic effects, which are independent of BRAF^V600^ inhibition^49^. To evaluate a possible relationship between Spry1 expression and ROS production in response to BRAFi treatment, Mel 272, Mel 599, and Mel 611 parental cell lines, and their respective Spry1^KO^ clones, were treated with vemurafenib 1 and 2 μM for 72 h. As shown in Fig. 7a, Spry1 protein was partially reduced in parental cell lines. We then assessed ROS levels and found that, consistent with published data^49^, vemurafenib treatment led to a concentration-dependent increase in ROS production, which was further potentiated in Spry1^KO^ clones (Fig. 7b).

As expected, Mel 599 and Mel 611 cells resulted responsive to vemurafenib, but a most significant and dose-dependent increase in the percentage of apoptotic cells was measured in their SPRY1^KO^ clones (Fig. 7c, Supplementary Figs. S6 and 7). Similar results were observed with the MEKi trametinib (Supplementary Fig. 8). Of note, 72 h after vemurafenib treatment, a stronger rebound of ERK1/2 activity was observed in Mel 599 and Mel 611 Spry1^KO^ clones compared with parental cells (Fig. 7d), which was likely due to a lack of a negative feedback mechanism^30^. Furthermore, when we probed the levels of γH2AX, a marker of DNA damage, we observed that γH2AX induction was markedly enhanced in Mel 599 and Mel 611 Spry1^KO^ clones (Fig. 7d). These data indicated that, following BRAFi treatment, the extent of pERK1/2 rebound, and thereby DNA damage, might depend on Spry1 expression levels.

As shown in Fig. 7e and in Supplementary Fig. 9, vemurafenib treatment significantly promoted a dose-dependent apoptosis also in Mel 272 Spry1^KO^ clone 4, but at a later time point. Notably, we confirmed that apoptosis associated with markedly increased of ERK1/2 and γH2AX phosphorylation levels in Spry1^KO^ cells (Fig. 7f). In addition, higher concentrations of vemurafenib significantly augmented the percentage of apoptotic cells in Mel 272 Spry1^KO^ clone 4, but not in parental cells (Supplementary Fig. S10), thus indicating that complete Spry1^KO^ was required to potentiate vemurafenib effects in BRAF^V600^-mutant CM cells. The preferential antitumor effect of vemurafenib on Spry1^KO^ cells was also observed in Mel 272 isogenic tumor xenografts. In fact, vemurafenib treatment was effective in further reducing the in vivo growth of xenografts from Mel 272 Spry1^KO^ clone 4 (Fig. 7g, h). In contrast, the difference in tumor volume between vemurafenib-treated or untreated Mel 272 tumors was not significant throughout the entire course of the experiment (Fig. 7g, h). Consistent with in vitro data, protein analyses of tumor tissues revealed that vemurafenib treatment significantly increased ERK1/2 phosphorylation, as well as γH2AX levels, in tumors from Mel 272 Spry1^KO^ clone 4 cells (Fig. 7i).

## Discussion

In the present study, we demonstrated, for the first time, that SPRY1 contributes to an “oncogenic” background in BRAF^V600E^-mutant CM. In fact, Spry1^KO^ reduced cell viability, delayed cell cycle progression, and increased apoptotic rate of BRAF^V600^-mutant CM cells. Furthermore, Spry1^KO^ decreased BRAF^V600^-mutant CM cell migration with concomitant reduction of several EMT-markers. Effects of Spry1^KO^ were even more dramatic in vivo, as it strongly reduced the tumor-forming capabilities of BRAF^V600^-mutant CM cells in xenograft assays. These findings are in agreement with other studies that showed that Spry1 suppression led to reduction in EMT in breast cancer^8^ and colorectal cancer^50^, and potently inhibited cell proliferation and survival of embryonal rhabdomyosarcoma subtype tumors^29^. EMT has been increasingly recognized as a crucial event in progression of BRAF^V600^-mutant CM^40^, as well as in resistance to targeted therapy^51^, and is usually associated with a high expression of MMP-2 (refs. ^40,52–54^). Although the precise molecular mechanism linking Spry1 to EMT needs further investigation, Spry1 expression appears to be critical for tumor induction, maintenance, and progression, and may potentially represent a novel vulnerability of CM harboring BRAF mutations.

Spry1^KO^ seems to affect BRAF^V600^-mutant CM irrespective of its genomic background. However, the mutational background of CM parental cells might determine some different responses to Spry1^KO^. For instance, CCND1 expression in Mel 599 parental cells may be related to the aberrant activity of β-catenin^36^. Consistently, Spry1^KO^ decreased β-catenin activation, and this effect coincided with a lower CCND1 expression. Although β-catenin has been reported to induce Spry2 expression in colon cancer^55^, no data about a direct interaction between Spry1 and β-catenin in BRAF^V600^-mutant CM were available up to now. Therefore, the Spry1/β-catenin/CCND1 signaling axis needs to be explored in future studies. In addition to the activating β-catenin mutation, Mel 599 cells harbored a splice-site mutation in TP53 gene that resulted in no detectable protein. Nonetheless, Spry1^KO^ enhanced basal apoptosis also in Mel 599 cells, indicating that modulators of apoptosis might be cell specific.

Our data showed that Spry1^KO^ decreased cell proliferation with the concomitant increase of ERK1/2 phosphorylation both in vitro and in vivo. Although MAPK/ERK activation usually promotes uncontrolled cellular growth and proliferation in CM, MAPK signaling must be finely tuned since it cannot be tolerated at supraphysiologic levels. Along this line, a recent study showed that ERK1/2 overexpression was toxic in CM cell lines that carried BRAF^V600^ mutation^56^. Here, we provide the evidence that Spry1^KO^ not only increased the phosphorylation of ERK1/2, but also strongly activated p38, which is known to be induced by cellular stress, including oxidative stress that usually results from excessive ROS production^57^. ROS appear a double-edged sword in tumor cells, since low to modest ROS levels sustain cancer cell proliferation and survival, whereas excessive ROS production causes cell cycle arrest and apoptosis^58^. Our study demonstrated that Spry1^KO^ increased the basal levels of intracellular ROS, and reduced the expression of the anti-oxidative response genes DnaJA4 and DnaJC15. DnaJA4 has been described as co-chaperone for the ATPase activity of Hsp70, and plays a major role in protecting stressed cells from apoptosis^59^, whereas DnaJC15 represents an endogenous mitochondrial repressor of the respiratory chain which expression is required to lower basal levels of ROS^60^. Altogether, these data lead us to speculate that the canonical function of Spry1 to dampen MAPK/ERK signaling is required for limiting oncogenic stress in BRAF^V600^ mutant CM.

**Fig. 8:**
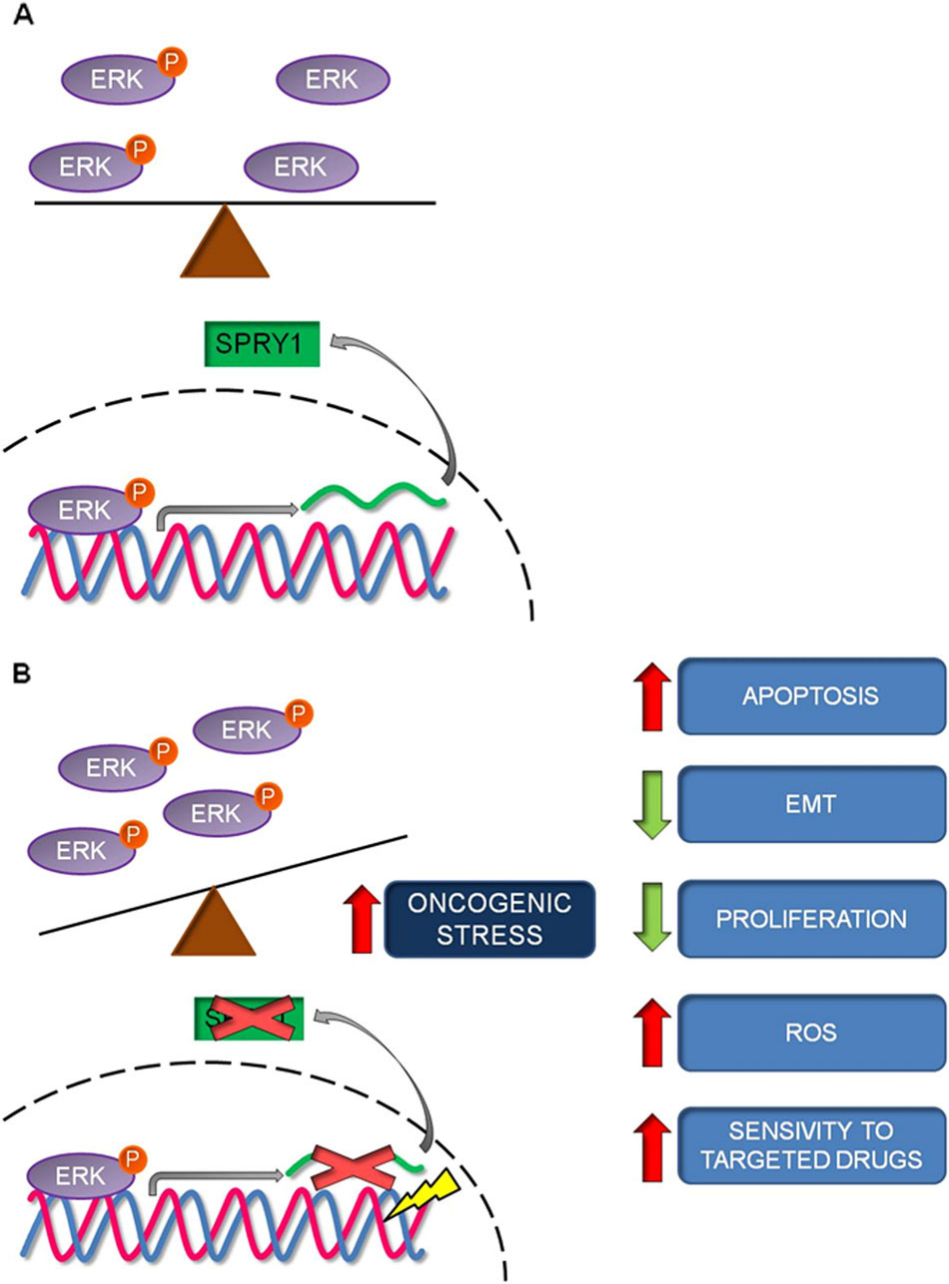
Proposed model for the contribution of Spry1 in BRAF^V600^-mutant CM. **a** Spry1, as a negative regulator of ERK1/2, presumably regulates the equilibrium between active and inactive ERK1/2 molecules. **b** Disruption of this feedback loop induces a robust ERK1/2 rebound that leads to oncogenic stress. Induction of ERK1/2 hyperactivation results in a significant decrease in cell proliferation and tumor migration. In addition, a strong ERK1/2 rebound increases oxidative stress and DNA damage, renders cells more susceptible to apoptosis, and sensitizes tumors to the treatment with targeted drugs.

Therapeutic targeting of MAPK signaling relieves MAPK-dependent feedback inhibition of negative regulators of ERK1/2, such as Spry1, thus inducing RAS activation and rebound of MAPK pathway^30^. Here, we found that the treatment with the BRAFi vemurafenib did not completely reduced Spry1 expression in the parental BRAF^V600^ mutant CM cells. Furthermore, although BRAFi significantly elevated oncogenic stress in the vemurafenib-sensitive parental BRAF^V600^ mutant CM cell lines, oxidative stress, ERK1/2 rebound, DNA damage, and apoptosis were further augmented upon Spry1^KO^. In the particular context of vemurafenib-resistant BRAF^V600^ mutant CM cells, the decreased Spry1 expression levels observed upon vemurafenib treatment could be not sufficient to induce a strong ERK1/2 rebound; thus, a full knockout of SPRY1 protein may be necessary to create a toxic state that renders treated cells more vulnerable to cell death. According with this hypothesis, we demonstrated that Spry1^KO^ was sufficient to sensitize the constitutively-resistant Mel 272 cells to vemurafenib antitumor activity both in vitro and in vivo. Therefore, the ability of Spry1 to regulate BRAF^V600^ mutant CM cell phenotypes is likely to be more complex than we have uncovered so far (Fig. 8).

In summary, this study provides deeper insights into BRAF^V600^-mutant CM biology, and suggest that at least 15% of mutant CM patients harboring BRAF^V600^ mutation might benefit from therapeutic approaches (i.e. RNAi-based therapies, antibodies) that specifically target Spry1 or other molecules whose pharmacological inhibition phenocopies the effects of Spry1^KO^. Future experiments will address in more detail the specific requirement of Spry1 for CM. In particular, it will be of interest to examine Spry1 expression levels in CM with mutant NRAS or wild type for both BRAF and NRAS mutations in order to better define what subpopulation of CM patients most likely benefit from a therapy with Spry1 inhibitors.

## Materials and methods

### Cells cultures and reagents

Cell cultures were established from metastatic lesions surgically removed from CM patients who did not underwent prior BRAFi therapy, who were referred to the National Cancer Institute of Aviano (Italy), as previously described^61^. Cell cultures were grown in RPMI-1640 Medium, supplemented with 2 mM l-glutamine (Sigma-Aldrich) and 10% heat-inactivated fetal calf serum (FCS, Lonza). The identity of paired Spry1^KO^ clones and parental cells was confirmed by short tandem repeat profiling using the Power Plex 1.2 kit (Promega). All cells were free of mycoplasma contamination as tested by PlasmoTest (Invivogen). Stock solutions of UO126 (Sigma), vemurafenib (PLX-4032, SeleckBio), and trametinib (GSK1120212, SeleckBio) were prepared in cell-culture grade DMSO (Sigma) as per the manufacturer’s indications. The study was approved by the Internal Review Board of the Centro di Riferimento Oncologico, IRCCS-National Cancer Institute, Aviano, Italy (IRB number 07-2017).

### BRAF, β-catenin, and p53 status

The mutational status of BRAF^V600^ (exon 15) was determined as previously described^62^, whereas the β-catenin^A121G^ point mutation was verified by allele-specific quantitative PCR (qPCR). Forward primer (5′-TTGATGGAGTTGGACATGGC-3′) was common to wild type (wt) and mutated (mut) sequences, whereas two different reverse primers with substitution of a single base at the end of the primer (reverse wt 5′-TCAGAGAAGGAGCTGTGGT-3′ and reverse mut 5′-TCAGAGAAGGAGCTGTGGG-3′) were designed to amplify wt or mut allele, respectively. QPCR reactions for wt and mut alleles were run in parallel on 10 ng of genomic DNA in a final volume of 20 μL SYBR-Green Universal Master Mix (ThermoFisher Scientific) at 95 °C for 10 min, followed by 45 cycles at 95 °C for 15 s and at 60 °C for 1 min, and dissociation performed at 95 °C for 15 s, 60 °C for 20 s, and 95 °C for 15. Known amounts of wt and mut DNA molecules were used to generate absolute standard curves. The copy number of wt and mut alleles was established by extrapolation from the standard curves. The percentage of mut allele was defined as the ratio between mut DNA molecules and the sum of wt and mut DNA molecules. The mutation within splicing site at the exon–intron 9 junction of TP53 gene was verified by Sanger sequencing using the following primers: TP53_forw, 5′-ACTGCCCAACAACACCAGCTCCT-3′ and TP53_rev, 5′-CATCACTGCCCCCTGATGGCAAA-3′.

### Generation of Spry1^KO^ BRAF^V600^-mutant CM clones

To perform genome editing, two guide sequences targeting SPRY1 (gRNA#1: CACTGCTCCAATGACGACGAAGG, and gRNA#2: CCAATGACGACGAAGGGGATTCC) were designed in the common coding exon (Supplementary Fig. S11) based on high target specificity and low number of off-target sites, as determined using the online CRISPR Design tool available at http://crispr.dbcls.jp/. Complementary oligonucleotides containing cloning overhangs were synthesized at Sigma, annealed, and the obtained double stranded oligonucleotide was cloned into the pSpCas9(BB)-2A-GFP (PX458) plasmid, kind gift from Feng Zhang (Addgene plasmid # 48138), as per the inventor’s protocol^63^. Plasmids were then transfected into CM cells using Lipofectamine 3000 reagent (ThermoFisher Scientific) following the manufacturer’s instructions. Two days after transfection, GFP-positive CM cells were sorted using FacsARIA III (Beckton Dickinson) and plated as single clones in 96-well plates. Clones were cultured for 2–3 weeks and analyzed for successful Spry1^KO^ by Sanger sequencing and western blotting. Validated Spry1^KO^ clones were then amplified and stored.

### RNA-seq

RNA-Seq libraries preparation was performed as previously described^64^. The raw sequence files generated (.fastq files) underwent quality control analysis using FASTQC (http://www.bioinformatics.babraham.ac.uk/projects/fastqc/) and adapter sequences were removed using Trimmomatic version 0.38 (ref. ^65^). Filtered reads were aligned on human genome (assembly hg38) considering genes present in GenCode Release 29 (GRCh38.p12) using STAR v2.5.3a (ref. ^66^) using standard parameters. Quantification of expressed genes was performed using HTSeq-count^67^ and differentially expressed genes were identified using DESeq2 (ref. ^68^). A given mRNA was considered expressed when detected by at least ≥10 raw reads. Differential expression was reported as |fold change|(FC) ≥ 1.5 along with associated adjusted *p* value ≤0.05 computed according to Benjamini–Hochberg. The RNA-seq raw data are publicly available in ArrayExpress repository under accession #E-MTAB-7886.

### Functional analysis

Functional and interaction network analysis was performed with IPA (www.ingenuity.com; Qiagen). Functional analysis on “molecular and cellular functions” category and canonical pathway investigation were carried out, calculating the likelihood that the association between our RNA dataset and a specific function or pathway is due to random choice and it is expressed as a *p* value calculated using the right-tailed Fisher’s exact test. The activation *Z*-score - is used to infer likely activation states of enriched pathways and functions, based on comparison with a model that assigns random regulation directions.

### Western blot analysis

Whole-cell lysate preparation and western blot were performed as previously described^69^. Detailed description of the used antibodies is reported in Supplementary Table 3. Images were captured and analyzed using the Chemidoc XRS + system (Bio-Rad). Expression levels were quantified using the ImageLab imaging software (Bio-Rad).

### Quantitative RT-PCR analysis

Real-time quantitative RT-PCR analyses were performed as described^70^ using Power SYBR-Green Master Mix (ThermoFisher Scientific). Primers sets used are listed in Supplementary Table 4. The absolute copy number of cDNA of target genes and of the reference gene β-actin was measured in each sample from standard curves. The number of target gene cDNA molecules in each sample was normalized to the number of cDNA molecules of β-actin.

### Spry1 intracellular localization

Analysis of Spry1 localization was performed by multispectral imaging flow cytometry. Samples were fixed with 2% PFA and permeabilized with cold methanol. After wash with phoshpate-buffered saline (PBS) containing 0.5% bovine serum albumin, cells were incubated with anti-Spry1 antibody (Anti-Spry1 (D9V6P) Cell Signaling Technology) at RT for 30 min. After two washes, cells were incubated for 30 min with PE-anti rabbit secondary antibody 1:100 (111-116-144) (Jackson ImmunoReseach) and the vital nuclear dye DRAQ5 (DR50200) (Alexis Biochemicals). 3 × 10^4^ cells/sample were acquired with Image-Stream X (Amnis, Millipore) using the INSPIRE software (Amnis, Millipore).

### Wound healing assay

Exponentially growing cells were seeded on 48-well plates to create a dense monolayer, and cultured until they reached confluence. A straight scratch was performed with using a sterile pipette tip. Cells were washed and incubated with RPMI-1640 medium. Time course analysis was carried out by the LEICA Time-lapse System.

### XCELLigence analysis

xCELLigence RTCA SP Station and Analyzer (ACEA Biosciences) was used for real-time cell growth analysis. The xCELLigence system was connected and tested by a resistor plate before the RTCA single plate station was placed inside the incubator at 37 °C and 5% CO_2_. 5 × 10^3^ cells were seeded on wells of a 96-well plate, and impedance of the wells was measured for 96 h. Every hour, cellular growth was measured and recorded as “doubling time” (DT). The amount of cell growth was analyzed and plotted using the RTCA Software (ACEA Biosciences).

### Cell cycle

CM cells were seeded into six-well plates (2 × 10^5^ cells per well) and cell cycle was analyzed by an FC500 Flow Cytometer (Beckman Coulter) after staining with SYTOX® AADvanced™ Dead Cell Stain kit (ThermoFisher Scientific) according to the protocol. Cell cycle analyses were performed using MultiCycle software (Phoenix flow systems).

### Reactive oxygen species

CM cells were seeded into six-well plates (2 × 10^5^ cells per well). ROS production was evaluated using ROS-ID® Superoxide Detection Kit (ENZ-51012; Enzo Life Sciences). Cells were washed with 1× wash buffer, and then resuspended in Superoxide Staining Solution by incubation at 37 °C for 30 min. Fluorescence was evaluated on a FC500 flow cytometer (Beckman Coulter).

### Apoptosis detection

For apoptosis evaluation cells were seeded into six-well plates at a density of 2 × 10^5^ cells/well. Apoptosis detection was performed through the analysis of Annexin V/PI staining (Roche, 11 988 549 001). Flow cytometric analyses were performed on an FC500 flow cytometer (Beckman Coulter).

### Animals

Six-week-old female athymic nude/nude mice, weighing approximately 23–25 g, were purchased from Envigo. Animal health was monitored daily by observation and sentinel animal blood sample analysis. BRAF-mutant CM cells and Spry1^KO^ clones (1.5 × 10^6^ cells/animal) were injected in 100 μL PBS subcutaneously in the flank region. Animals were examined daily, and tumor development was measured every 2–3 days for 32 days. For treatment with BRAFi, when tumors were palpable (diameter ≥0.1 cm), animals were divided into two groups and treated every day with vemurafenib (intraperitoneal injection, 30 mg/kg) or vehicle. Tumor volume was measured every 2–3 days for 21 days. Animals were sacrificed by CO_2_ overdose. All the in vivo studies were approved by the Institutional Ethics Committee of the CRO of Aviano and the Italian Ministry of Health (no. 788/2015/PR).

### Statistical analysis

All data were expressed as the mean ± SD from at least three measurements on randomized samples. The sample size for the in vitro and in vivo experiments was established based on the experience of the single researches with the techniques employed. Since the variance between groups was similar and values were normally distributed, the statistical significance of the differences for the in vitro and in vivo experiments was determined by the two-sided *t*-test. For the in vivo experiments mice were randomly assigned to treatment groups. Differences were considered statistically significant when *p* values <0.05.

## Supplementary information

Supplementary Figure Legends_clean version

Supplementary Table 1

Supplementary Table 2

Supplementary Table 3

Supplementary Table 4

Supplementary Figure S1

Supplementary Figure S2

Supplementary Figure S3

Supplementary Figure S4

Supplementary Figure S5

Supplementary Figure S6

Supplementary Figure S7

Supplementary Figure S8

Supplementary Figure S9

Supplementary Figure S10

Supplementary Figure S11
