# Supplementary material for: Loss of Spry1 reduces growth of BRAFV600-mutant cutaneous melanoma and improves response to targeted therapy
Source: Cell Death Dis. 2020 May 22;11(5):392. doi: 10.1038/s41419-020-2585-y (PMC7244546; doi:10.1038/s41419-020-2585-y)
Supplement: Supplementary file 1 — Supplementary Figure Legends_clean version [file 41419_2020_2585_MOESM1_ESM.doc]

**FIGURE LEGENDS_SUPPLEMENTARY**

**Figure S1.** Representative results of Sanger sequencing studies showing the presence of a point mutation in the splice acceptor site in TP53 intron 9 in Mel 599 cells. Red boxes indicate nucleotide variants.

**Figure S2.** **a** Representative results of Sanger sequencing studies showing the presence of a nucleotide substitution (121A>G) into the exon 3 of the β-catenin gene in Mel 599 cells. Red boxes indicate nucleotide variants. **b** Western blot analysis of total and Ser33/37/Thr41 non-phospho (active) β-catenin (non-p-β-catenin) in six BRAF^V600^-mutant CM cell lines. β-tubulin was used as loading control.

**Figure S3.** Spry1 expression in Mel 272 BRAF^V600^-mutant CM cell line and respective Spry1^KO^ clone 4. β-tubulin was used as a loading control.

**Figure S4.** Representative results of Sanger sequencing studies showing the presence of BRAF^V600K^ in Mel 272 cells. Red boxes indicate nucleotide variants.

**Figure S5.** Phosphorylated protein levels form Fig. 3i presented normalized to β-tubulin. For the quantification analysis, the sum of the density of bands under study was calculated, and normalized to the amount of β-tubulin. After normalization with β-tubulin, changes in protein phosphorylation in Spry1^KO^ clones were calculated relative to the parental basal level. **a** Expression ratio of pERK1/β-tubulin. **b** Expression ratio of pERK2/β-tubulin. **c** Expression ratio of pp38/β-tubulin. Shown are means of three independent experiments ± SD. Statistically significant differences were indicated: **, *p* <0.01.

**Figure S6.** Percentage of apoptotic cells of Mel 599 cell line and its respective clone following treatment with vemurafenib at the indicated concentrations for 3 days.

**Figure S7.** Percentage of apoptotic cells of Mel 611 cell line and its respective clone following treatment with vemurafenib at the indicated concentrations for 3 days.

**Figure S8.** The effect of tramentinib in Mel 599 and Mel 611 parental cells and their respective Spry1^KO^ clones. Spry1^KO^ cells have significantly higher percentage of cells undergoing apoptosis (assessed by Annexin V/PI staining) after treatment with tramentinib 4 nM for 3 days. Shown are means of three independent experiments ± SD. Statistically significant differences were indicated: **, *p* <0.01.

**Figure S9.** Percentage of apoptotic cells of Mel 272 cell line and its respective clone following treatment with vemurafenib at the indicated concentrations for 3 days.

**Figure S10.** The effect of vemurafenib in Mel 272 parental and Spry1^KO^ cells. Spry1^KO^ cells have significantly higher percentage of cells undergoing apoptosis (assessed by Annexin V/PI staining) after treatment for 5 days with increasing doses of vemurafenib (1 μM, 2 μM, 4 μM). Shown are means of three independent experiments ± SD. Statistically significant differences were indicated: *, *p* <0.05, **, *p* <0.01.

**Figure S11.** Generation of Spry1^KO^ using CRISPR/Cas9 technology. The gRNA target sequences and PAM domains are indicated by black and red underlining, respectively.
