# Supplementary material for: Loss of Spry1 reduces growth of BRAFV600-mutant cutaneous melanoma and improves response to targeted therapy
Source: Cell Death Dis. 2020 May 22;11(5):392. doi: 10.1038/s41419-020-2585-y (PMC7244546; doi:10.1038/s41419-020-2585-y)
Supplement: Supplementary file 2 — Supplementary Table 1 [file 41419_2020_2585_MOESM2_ESM.doc]

Supplementary Table S1. Sequencing of BRAF V600 codon of CM cells

| Mel | BRAF status | Braf mutation |
| --- | --- | --- |
| 272 | mut/mut^1^ | V600K (AAG)^2^ |
| 380 | wt/mut | V600E (GAG) |
| 514 | wt/mut | V600K (AAG) |
| 593 | wt/mut | V600E (GAG) |
| 599 | wt/mut | V600E (GAG) |
| 611 | wt/mut | V600E (GAG) |

^1^ wt = wild tipe sequence; mut = mutated sequence

^2^ sequence of the mutated codon and respective amino acid substitution
