# Supplementary material for: Loss of Spry1 reduces growth of BRAFV600-mutant cutaneous melanoma and improves response to targeted therapy
Source: Cell Death Dis. 2020 May 22;11(5):392. doi: 10.1038/s41419-020-2585-y (PMC7244546; doi:10.1038/s41419-020-2585-y)
Supplement: Supplementary file 3 — Supplementary Table 2 [file 41419_2020_2585_MOESM3_ESM.doc]

| Supplementary Table S2. List of genes commonly modulated in Mel 599 and Mel 611 Spry1^KO^ clones | | | | |
| --- | --- | --- | --- | --- |
|
|  |  |  |  |  |
| **Gene** | **FC_Mel 599 SPRY1^KO^ clone 9 vs Parental** | **padj** | **FC_Mel 611 SPRY1^KO^ clone 4 vs Parental** | **padj** |
| DNAJA4 | **-4.414** | 4.55E-06 | **-124.396** | 3.08E-79 |
| AEBP1 | **-111.704** | 8.63E-34 | **-13.496** | 1.10E-09 |
| TTC39A | **-7.702** | 4.49E-15 | **-12.780** | 1.54E-06 |
| ZNF736 | **-3.663** | 5.67E-55 | **-11.017** | 4.75E-33 |
| SQRDL | **-3.128** | 5.91E-03 | **-9.515** | 2.10E-04 |
| SERPINF1 | **-1.603** | 7.88E-13 | **-8.886** | 2.43E-105 |
| IFITM2 | **-23.611** | 8.22E-16 | **-8.279** | 6.04E-15 |
| ZNF737 | **-22.228** | 7.00E-27 | **-7.002** | 1.38E-05 |
| WFDC1 | **-119.043** | 4.22E-99 | **-5.734** | 9.14E-14 |
| TSC22D3 | **-4.070** | 6.60E-41 | **-5.346** | 3.13E-22 |
| TF | **-2.003** | 2.93E-02 | **-5.238** | 1.01E-05 |
| ENPP2 | **-5.875** | 3.01E-11 | **-4.854** | 4.59E-08 |
| LINC00906 | **-7.068** | 2.11E-17 | **-4.347** | 8.15E-03 |
| BAALC | **-9.626** | 1.12E-05 | **-4.267** | 2.46E-02 |
| RLBP1 | **-5.496** | 1.97E-04 | **-4.216** | 7.71E-09 |
| GLUL | **-2.325** | 1.01E-126 | **-4.097** | 3.95E-66 |
| ZIK1 | **-4.498** | 7.93E-05 | **-3.986** | 8.11E-07 |
| LINC00944 | **-4.649** | 4.33E-04 | **-3.934** | 1.81E-06 |
| LINC01057 | **-22.957** | 2.15E-10 | **-3.852** | 2.51E-02 |
| BMP8B | **-1.950** | 3.40E-04 | **-3.811** | 1.33E-03 |
| LIPE | **-9.136** | 2.64E-08 | **-3.555** | 9.37E-04 |
| RP4-718J7.4 | **-1.524** | 4.24E-02 | **-3.541** | 1.32E-08 |
| PLTP | **-114.558** | 3.56E-118 | **-3.486** | 2.17E-10 |
| DNAJC15 | **-37.172** | 2.98E-165 | **-3.428** | 5.44E-17 |
| CDH1 | **-2.236** | 8.01E-82 | **-3.400** | 2.40E-08 |
| ADAMTS2 | **-31.435** | 9.55E-121 | **-3.345** | 2.12E-03 |
| IRF4 | **-1.907** | 1.78E-49 | **-3.317** | 4.96E-45 |
| RNU1-85P | **-3.675** | 4.37E-15 | **-3.317** | 1.36E-02 |
| LRRC2 | **-1.718** | 3.95E-02 | **-3.282** | 1.50E-03 |
| LINC01443 | **-2.917** | 1.76E-128 | **-3.233** | 6.77E-31 |
| ENPP5 | **-5.723** | 8.95E-04 | **-3.220** | 1.06E-02 |
| SDC4 | **-2.280** | 1.37E-23 | **-3.171** | 3.36E-15 |
| ZNF585B | **-5.618** | 5.76E-11 | **-3.052** | 4.05E-04 |
| TYRP1 | **-1.561** | 1.16E-22 | **-3.038** | 9.19E-89 |
| ZFAND4 | **-4.607** | 2.77E-12 | **-2.988** | 5.64E-03 |
| PCDHGB5 | **-4.392** | 1.69E-11 | **-2.976** | 6.98E-05 |
| PNMAL1 | **-7.130** | 5.57E-06 | **-2.952** | 1.11E-17 |
| PAEP | **-3.037** | 4.18E-08 | **-2.941** | 9.10E-07 |
| ZNF418 | **-7.123** | 4.80E-05 | **-2.917** | 2.09E-03 |
| TNS1 | **-3.634** | 3.52E-07 | **-2.869** | 5.21E-23 |
| EGLN3 | **-12.532** | 1.78E-127 | **-2.847** | 2.00E-02 |
| ZNF702P | **-11.191** | 2.33E-14 | **-2.810** | 6.25E-03 |
| CX3CL1 | **-4.126** | 4.40E-07 | **-2.730** | 5.27E-09 |
| ARMCX1 | **-511.714** | 6.63E-67 | **-2.714** | 5.71E-08 |
| CCDC109B | **-2.346** | 5.69E-07 | **-2.673** | 5.89E-07 |
| GATM | **-3.152** | 3.05E-05 | **-2.663** | 4.11E-02 |
| METTL7A | **-6.774** | 9.91E-45 | **-2.490** | 4.18E-02 |
| IGFBP3 | **-3.388** | 2.60E-13 | **-2.470** | 1.63E-03 |
| LOXL2 | **-1.578** | 3.40E-02 | **-2.461** | 1.52E-08 |
| FAM129A | **-12.137** | 1.37E-16 | **-2.419** | 7.37E-04 |
| ZNF585A | **-20.968** | 2.71E-17 | **-2.398** | 2.11E-03 |
| QPRT | **-2.246** | 2.94E-47 | **-2.389** | 4.71E-04 |
| ROPN1 | **-1.877** | 6.53E-03 | **-2.370** | 1.73E-03 |
| QDPR | **-2.076** | 2.67E-36 | **-2.355** | 7.25E-18 |
| LDHAP4 | **-2.131** | 9.13E-06 | **-2.340** | 6.99E-04 |
| CYB561 | **-2.580** | 1.29E-05 | **-2.314** | 2.25E-04 |
| AIF1L | **-12.546** | 2.31E-121 | **-2.304** | 2.60E-10 |
| SPINT1 | **-2.316** | 2.13E-02 | **-2.300** | 5.02E-04 |
| RP11-4B16.4 | **-1.742** | 1.37E-09 | **-2.273** | 7.98E-09 |
| DANCR | **-2.403** | 1.31E-39 | **-2.256** | 5.60E-13 |
| LUM | **-2.535** | 1.07E-07 | **-2.250** | 2.87E-21 |
| NCALD | **-2.187** | 1.92E-32 | **-2.222** | 1.22E-16 |
| SMARCA2 | **-1.597** | 3.35E-17 | **-2.214** | 8.06E-15 |
| GYG2 | **-1.774** | 9.59E-24 | **-2.211** | 1.37E-23 |
| C8orf88 | **-2.587** | 6.58E-16 | **-2.207** | 1.26E-02 |
| RP11-458J1.1 | **-1.663** | 9.41E-04 | **-2.186** | 4.75E-03 |
| GAS1 | **-1.849** | 1.08E-05 | **-2.183** | 5.06E-04 |
| SMAD7 | **-2.365** | 9.01E-16 | **-2.181** | 7.94E-06 |
| FSTL1 | **-3.541** | 3.58E-202 | **-2.178** | 3.52E-43 |
| S100A13 | **-2.365** | 7.39E-46 | **-2.161** | 4.69E-20 |
| CA14 | **-26.014** | 1.10E-30 | **-2.150** | 7.03E-10 |
| S100A16 | **-2.362** | 9.18E-03 | **-2.148** | 3.16E-17 |
| C17orf75 | **-2.561** | 3.51E-20 | **-2.146** | 5.02E-04 |
| PCLO | **-5.751** | 9.79E-09 | **-2.133** | 9.81E-03 |
| MMP2 | **-16.417** | 7.47E-26 | **-2.124** | 2.52E-09 |
| GPM6A | **-15.502** | 1.41E-60 | **-2.084** | 1.64E-16 |
| F8A1 | **-2.889** | 5.52E-07 | **-2.064** | 1.84E-02 |
| C4orf3 | **-112.800** | 4.32E-42 | **-2.063** | 5.00E-15 |
| MAGEH1 | **-2.460** | 2.31E-29 | **-2.053** | 1.16E-09 |
| DYRK4 | **-1.980** | 7.35E-13 | **-2.039** | 2.89E-03 |
| GPR85 | **-1.655** | 8.78E-09 | **-2.033** | 3.89E-02 |
| DPCD | **-1.774** | 2.12E-10 | **-2.024** | 1.63E-05 |
| ANG | **-1.905** | 2.57E-04 | **-2.019** | 7.48E-03 |
| IRX3 | **-1.977** | 2.52E-15 | **-1.994** | 1.06E-02 |
| RGS3 | **-2.674** | 2.96E-14 | **-1.970** | 3.86E-02 |
| MTRNR2L10 | **-17.463** | 9.07E-73 | **-1.969** | 4.62E-02 |
| NDUFC1 | **-1.706** | 1.81E-17 | **-1.966** | 8.08E-11 |
| ZNF518B | **-1.505** | 2.53E-12 | **-1.958** | 3.09E-17 |
| RP11-49I11.1 | **-1.866** | 5.55E-03 | **-1.954** | 7.02E-03 |
| TIFA | **-1.835** | 4.89E-09 | **-1.946** | 9.33E-04 |
| PGAM1 | **-2.444** | 4.43E-55 | **-1.945** | 1.41E-15 |
| TMEM45A | **-2.006** | 6.51E-05 | **-1.936** | 3.03E-05 |
| RGS20 | **-1.583** | 2.68E-08 | **-1.931** | 7.22E-06 |
| TBC1D7 | **-1.560** | 9.39E-20 | **-1.925** | 6.86E-25 |
| HAUS1 | **-1.752** | 4.29E-15 | **-1.924** | 1.07E-07 |
| GINS2 | **-5.688** | 7.94E-115 | **-1.913** | 2.64E-07 |
| PHACTR1 | **-2.522** | 1.08E-59 | **-1.910** | 6.11E-13 |
| OPHN1 | **-2.141** | 4.77E-19 | **-1.906** | 4.92E-10 |
| ITGB3 | **-2.157** | 2.48E-04 | **-1.898** | 9.43E-05 |
| MAGEA6 | **-22.991** | 7.38E-18 | **-1.881** | 1.26E-11 |
| ZNF677 | **-8.282** | 4.01E-06 | **-1.877** | 2.32E-02 |
| RAB32 | **-2.026** | 6.58E-61 | **-1.873** | 1.02E-16 |
| GLA | **-1.713** | 2.40E-28 | **-1.866** | 5.73E-15 |
| TRAM1L1 | **-2.333** | 2.33E-07 | **-1.861** | 2.28E-02 |
| KDELC1 | **-3.371** | 1.67E-38 | **-1.856** | 7.95E-04 |
| CTSL | **-2.453** | 7.17E-123 | **-1.853** | 1.01E-23 |
| TMEM169 | **-3.661** | 7.20E-25 | **-1.848** | 6.36E-03 |
| TRIM63 | **-2.378** | 2.19E-37 | **-1.838** | 1.58E-06 |
| COPRS | **-2.041** | 6.11E-38 | **-1.837** | 4.94E-09 |
| MAGEA3 | **-11.642** | 5.81E-13 | **-1.834** | 2.44E-11 |
| PET100 | **-1.914** | 6.70E-10 | **-1.833** | 2.42E-03 |
| FH | **-1.647** | 7.67E-22 | **-1.831** | 2.13E-13 |
| CHCHD1 | **-1.615** | 1.92E-10 | **-1.827** | 1.33E-05 |
| TUBB2B | **-2.236** | 9.17E-14 | **-1.826** | 1.10E-02 |
| SEC11C | **-2.669** | 6.86E-98 | **-1.825** | 3.17E-17 |
| SAP30 | **-1.579** | 9.52E-08 | **-1.824** | 1.90E-04 |
| MRAS | **-1.903** | 5.27E-38 | **-1.818** | 2.19E-12 |
| ATPIF1 | **-1.583** | 6.31E-10 | **-1.814** | 2.99E-07 |
| COX14 | **-4.104** | 2.43E-46 | **-1.808** | 3.97E-04 |
| MYL6 | **-2.451** | 1.24E-100 | **-1.801** | 1.73E-24 |
| ARHGEF9 | **-2.258** | 8.16E-44 | **-1.798** | 2.38E-12 |
| LINC00957 | **-3.353** | 5.45E-03 | **-1.797** | 4.68E-02 |
| TPGS2 | **-1.608** | 1.08E-25 | **-1.797** | 9.94E-19 |
| HIST1H2BM | **-1.635** | 8.33E-18 | **-1.797** | 2.70E-15 |
| GPR176 | **-2.729** | 7.31E-29 | **-1.795** | 2.90E-03 |
| HIST1H4H | **-2.341** | 3.18E-35 | **-1.792** | 5.47E-12 |
| TTC39C | **-2.947** | 7.77E-26 | **-1.788** | 1.43E-04 |
| MAP2K6 | **-2.337** | 2.97E-19 | **-1.787** | 4.33E-04 |
| CCDC167 | **-3.765** | 1.37E-60 | **-1.780** | 2.28E-05 |
| SOCS6 | **-1.542** | 4.04E-20 | **-1.770** | 1.11E-14 |
| ZNF141 | **-71.817** | 9.24E-31 | **-1.770** | 2.45E-04 |
| COMMD3 | **-1.730** | 1.53E-08 | **-1.765** | 1.78E-03 |
| ERAL1 | **-1.988** | 5.17E-21 | **-1.763** | 5.77E-05 |
| PARD6G | **-2.087** | 1.64E-26 | **-1.758** | 1.57E-07 |
| NUDT2 | **-1.933** | 4.31E-15 | **-1.758** | 2.80E-03 |
| UQCRQ | **-1.819** | 6.22E-41 | **-1.748** | 5.92E-15 |
| PIGS | **-1.508** | 1.18E-11 | **-1.744** | 3.76E-09 |
| LSAMP | **-4.704** | 3.30E-79 | **-1.742** | 8.89E-16 |
| SKA2 | **-1.872** | 1.41E-25 | **-1.742** | 1.42E-08 |
| TMEM70 | **-1.588** | 5.55E-11 | **-1.733** | 3.23E-06 |
| PEX11B | **-1.842** | 5.10E-12 | **-1.717** | 2.63E-04 |
| TIMM8B | **-2.318** | 3.87E-37 | **-1.716** | 3.74E-05 |
| SGMS1-AS1 | **-1.681** | 1.98E-02 | **-1.716** | 1.03E-02 |
| MPP1 | **-1.841** | 7.93E-15 | **-1.715** | 3.80E-04 |
| MPC2 | **-2.023** | 8.53E-37 | **-1.715** | 5.75E-08 |
| VPS25 | **-2.180** | 1.17E-41 | **-1.709** | 9.27E-08 |
| UBL5 | **-2.887** | 6.31E-114 | **-1.702** | 2.68E-11 |
| CHFR | **-2.219** | 1.70E-33 | **-1.699** | 7.99E-06 |
| TMEM185A | **-1.573** | 1.14E-04 | **-1.694** | 7.05E-03 |
| AKIP1 | **-1.764** | 5.55E-14 | **-1.690** | 1.53E-04 |
| CD27-AS1 | **-4.035** | 2.47E-05 | **-1.688** | 1.12E-02 |
| GLRX2 | **-1.767** | 1.03E-11 | **-1.687** | 4.27E-03 |
| LINC00665 | **-3.460** | 1.02E-04 | **-1.684** | 1.71E-02 |
| TMEM256 | **-1.615** | 2.95E-05 | **-1.683** | 1.33E-02 |
| KLHL5 | **-1.545** | 9.10E-15 | **-1.677** | 1.89E-08 |
| AFAP1 | **-1.672** | 1.15E-08 | **-1.677** | 1.59E-07 |
| ATP6V0D1 | **-2.446** | 3.87E-32 | **-1.676** | 1.05E-08 |
| COA3 | **-1.661** | 1.19E-14 | **-1.674** | 1.98E-05 |
| CETN2 | **-1.909** | 1.12E-20 | **-1.674** | 6.28E-07 |
| DUSP23 | **-11.819** | 1.46E-23 | **-1.671** | 4.71E-03 |
| NME1 | **-2.626** | 1.76E-79 | **-1.669** | 1.29E-09 |
| MPZL1 | **-2.108** | 9.17E-72 | **-1.668** | 8.13E-18 |
| AKTIP | **-2.090** | 4.77E-12 | **-1.667** | 1.67E-03 |
| SLC25A20 | **-1.791** | 6.36E-08 | **-1.667** | 1.88E-03 |
| ZNF69 | **-21.470** | 2.45E-20 | **-1.662** | 2.24E-02 |
| EBP | **-2.590** | 9.42E-71 | **-1.660** | 9.05E-11 |
| PSMD10 | **-2.060** | 3.14E-50 | **-1.660** | 3.63E-11 |
| NRSN2 | **-2.199** | 8.15E-27 | **-1.658** | 1.17E-08 |
| SNAPIN | **-2.501** | 5.28E-32 | **-1.654** | 2.25E-04 |
| DTL | **-1.820** | 4.22E-35 | **-1.649** | 6.42E-11 |
| MAGEA12 | **-3.172** | 4.33E-07 | **-1.649** | 1.20E-07 |
| NDUFA2 | **-1.725** | 4.38E-15 | **-1.646** | 1.03E-04 |
| SMIM14 | **-1.755** | 1.15E-15 | **-1.641** | 2.59E-06 |
| SEC61G | **-2.161** | 4.25E-44 | **-1.640** | 1.89E-11 |
| XXYLT1 | **-1.945** | 3.47E-28 | **-1.632** | 1.27E-04 |
| S100A4 | **-5.029** | 1.53E-04 | **-1.632** | 6.74E-03 |
| ENOPH1 | **-1.523** | 3.16E-15 | **-1.631** | 4.22E-11 |
| PSPH | **-1.732** | 2.29E-16 | **-1.630** | 1.43E-09 |
| KIF6 | **-2.106** | 2.97E-06 | **-1.629** | 5.00E-03 |
| PKNOX2 | **-12.956** | 7.13E-25 | **-1.622** | 6.11E-04 |
| CHST3 | **-2.181** | 2.10E-39 | **-1.619** | 1.49E-06 |
| CAPN3 | **-5.518** | 5.79E-28 | **-1.616** | 2.28E-05 |
| COX8A | **-1.615** | 2.54E-13 | **-1.615** | 3.39E-10 |
| HIST1H2BE | **-14.637** | 1.28E-38 | **-1.614** | 3.39E-03 |
| CDC6 | **-1.996** | 2.47E-42 | **-1.613** | 2.64E-09 |
| CDK2AP1 | **-2.286** | 3.35E-88 | **-1.611** | 6.46E-15 |
| FAM111B | **-23.232** | 1.61E-22 | **-1.611** | 6.80E-06 |
| MAPKAPK2 | **-1.831** | 1.16E-36 | **-1.611** | 2.13E-07 |
| APOO | **-2.289** | 1.23E-26 | **-1.610** | 4.52E-05 |
| ATP5I | **-1.632** | 5.88E-17 | **-1.604** | 6.26E-09 |
| SMS | **-1.825** | 1.12E-39 | **-1.604** | 8.76E-15 |
| ZNF542P | **-19.662** | 1.02E-32 | **-1.602** | 4.29E-02 |
| SMIM20 | **-1.730** | 2.12E-08 | **-1.599** | 1.06E-03 |
| MET | **-2.213** | 9.46E-65 | **-1.599** | 6.48E-11 |
| DHFR | **-1.952** | 1.67E-33 | **-1.598** | 3.26E-07 |
| FBXO8 | **-1.944** | 1.01E-14 | **-1.594** | 4.48E-03 |
| MCTS1 | **-2.002** | 7.12E-33 | **-1.594** | 1.29E-06 |
| ORC1 | **-2.176** | 4.25E-43 | **-1.593** | 4.54E-05 |
| GAMT | **-1.614** | 7.19E-03 | **-1.590** | 5.54E-03 |
| TXNDC17 | **-2.738** | 8.34E-59 | **-1.588** | 8.34E-05 |
| S100A11 | **-1.672** | 2.61E-32 | **-1.588** | 3.09E-13 |
| TRIM48 | **-3.814** | 1.15E-81 | **-1.583** | 1.61E-10 |
| STAT3 | **-1.631** | 3.59E-21 | **-1.582** | 2.04E-09 |
| RAB28 | **-1.548** | 3.02E-08 | **-1.579** | 1.87E-04 |
| BAMBI | **-1.500** | 6.35E-28 | **-1.578** | 1.59E-12 |
| MGST3 | **-1.567** | 4.74E-29 | **-1.576** | 1.45E-10 |
| NEDD8 | **-2.278** | 3.56E-54 | **-1.572** | 2.13E-06 |
| UBE2T | **-3.184** | 1.06E-132 | **-1.572** | 4.30E-06 |
| TMEM14A | **-1.528** | 4.52E-10 | **-1.568** | 8.57E-06 |
| XPNPEP1 | **-1.792** | 6.08E-53 | **-1.568** | 9.52E-13 |
| FKBP9P1 | **-1.523** | 1.38E-02 | **-1.567** | 2.21E-02 |
| DISC1 | **-2.764** | 4.16E-70 | **-1.566** | 7.70E-03 |
| PNPO | **-1.995** | 1.69E-25 | **-1.565** | 1.62E-05 |
| NFU1 | **-1.513** | 3.71E-08 | **-1.565** | 6.57E-04 |
| TIMM8A | **-1.893** | 1.38E-09 | **-1.564** | 1.46E-02 |
| MCM10 | **-1.899** | 1.42E-25 | **-1.563** | 4.72E-05 |
| PSMC3IP | **-1.592** | 2.23E-06 | **-1.559** | 2.15E-02 |
| UPRT | **-1.501** | 7.29E-06 | **-1.557** | 2.23E-03 |
| ADH5 | **-2.035** | 1.02E-63 | **-1.555** | 6.83E-13 |
| HIRIP3 | **-1.593** | 4.48E-06 | **-1.555** | 1.80E-02 |
| PRDM7 | **-2.282** | 1.82E-30 | **-1.555** | 7.22E-07 |
| NDUFB6 | **-2.022** | 3.79E-30 | **-1.551** | 6.63E-05 |
| GK | **-1.652** | 2.80E-15 | **-1.551** | 4.87E-08 |
| SLBP | **-1.958** | 2.97E-47 | **-1.550** | 6.71E-09 |
| MFSD5 | **-1.836** | 8.26E-14 | **-1.549** | 4.58E-04 |
| YIPF1 | **-1.540** | 1.69E-10 | **-1.546** | 1.09E-04 |
| PRKAR2B | **-1.851** | 3.53E-14 | **-1.546** | 2.60E-05 |
| MAFF | **-1.554** | 1.27E-12 | **-1.545** | 4.28E-08 |
| SLAIN1 | **-4.173** | 2.82E-45 | **-1.544** | 4.62E-06 |
| ATP6V1H | **-1.824** | 7.60E-37 | **-1.542** | 6.83E-09 |
| ABCA1 | **-2.460** | 7.56E-04 | **-1.539** | 8.51E-06 |
| THBS1 | **-2.255** | 8.76E-03 | **-1.538** | 4.69E-08 |
| STAM | **-2.062** | 1.63E-48 | **-1.537** | 1.49E-07 |
| USMG5 | **-2.390** | 3.73E-72 | **-1.537** | 7.83E-08 |
| NKIRAS2 | **-1.982** | 5.89E-18 | **-1.534** | 3.05E-03 |
| CDKN2C | **-1.547** | 1.67E-11 | **-1.534** | 2.72E-05 |
| UBE2L6 | **-1.888** | 5.50E-15 | **-1.534** | 2.50E-03 |
| RPL26L1 | **-1.971** | 5.46E-17 | **-1.534** | 1.82E-03 |
| PJA1 | **-2.296** | 6.77E-33 | **-1.533** | 3.63E-04 |
| PRKCDBP | **-52.408** | 3.55E-28 | **-1.533** | 1.32E-04 |
| PSMD11 | **-1.520** | 1.38E-20 | **-1.528** | 1.08E-07 |
| MIEN1 | **-2.018** | 9.52E-23 | **-1.527** | 6.59E-04 |
| PDZD11 | **-2.389** | 1.00E-38 | **-1.521** | 4.20E-04 |
| MIF4GD | **-1.752** | 1.41E-06 | **-1.520** | 4.18E-02 |
| HINT1 | **-2.188** | 8.58E-75 | **-1.519** | 6.86E-12 |
| MAPRE2 | **-2.315** | 4.97E-38 | **-1.519** | 1.14E-04 |
| TXN | **-1.886** | 1.55E-34 | **-1.519** | 2.90E-08 |
| PXMP4 | **-2.053** | 4.14E-17 | **-1.518** | 6.95E-04 |
| COMMD9 | **-1.989** | 3.28E-20 | **-1.517** | 4.52E-04 |
| CD99L2 | **-1.547** | 2.41E-14 | **-1.516** | 4.93E-09 |
| VDR | **-2.808** | 4.45E-07 | **-1.512** | 5.70E-03 |
| PCGF6 | **-1.634** | 2.71E-08 | **-1.512** | 1.10E-02 |
| TMEM126A | **-1.547** | 1.93E-09 | **-1.512** | 2.39E-03 |
| PIR | **-2.339** | 4.59E-75 | **-1.508** | 8.08E-11 |
| SNRPD1 | **-2.047** | 3.02E-38 | **-1.508** | 6.79E-06 |
| RHNO1 | **-1.579** | 3.86E-11 | **-1.506** | 8.72E-04 |
| SYT11 | **-2.418** | 7.52E-76 | **-1.506** | 3.46E-03 |
| HIST1H1A | **-4.558** | 4.74E-104 | **-1.503** | 1.43E-02 |
| MAD2L1 | **-1.739** | 7.94E-32 | **-1.502** | 6.32E-09 |
| C1orf131 | **-1.854** | 6.98E-11 | **-1.502** | 4.19E-02 |
| MED8 | **-2.087** | 2.25E-33 | **-1.501** | 3.52E-05 |
| ARHGEF10L | **1.905** | 9.45E-10 | **1.515** | 3.97E-03 |
| CREBBP | **2.113** | 1.55E-09 | **1.517** | 1.86E-05 |
| SERINC2 | **5.691** | 2.27E-43 | **1.518** | 2.31E-02 |
| ZYX | **2.397** | 8.72E-22 | **1.519** | 6.58E-07 |
| RP11-107E5.3 | **3.529** | 4.23E-23 | **1.521** | 6.86E-03 |
| RP11-159D12.8 | **1.504** | 3.05E-04 | **1.523** | 1.77E-02 |
| SNORA74A | **11.125** | 1.48E-37 | **1.534** | 4.23E-03 |
| CRTC1 | **5.399** | 2.10E-36 | **1.541** | 6.93E-03 |
| AC058791.1 | **1.970** | 7.47E-22 | **1.541** | 2.35E-07 |
| CACNA1H | **4.316** | 4.35E-23 | **1.547** | 1.02E-03 |
| TSPAN14 | **1.713** | 3.27E-38 | **1.548** | 1.19E-10 |
| GS1-124K5.11 | **3.018** | 8.88E-12 | **1.549** | 1.74E-02 |
| ZNF277 | **1.585** | 6.76E-11 | **1.555** | 1.15E-07 |
| FRMD4A | **2.621** | 1.04E-39 | **1.560** | 2.53E-04 |
| UBAP1L | **2.150** | 1.24E-12 | **1.562** | 7.00E-04 |
| ANKMY1 | **3.343** | 7.26E-29 | **1.565** | 7.80E-04 |
| CFAP69 | **1.970** | 8.34E-04 | **1.566** | 3.97E-02 |
| FBRSL1 | **2.724** | 7.48E-16 | **1.568** | 3.86E-03 |
| FAM81A | **2.086** | 3.79E-16 | **1.577** | 2.29E-03 |
| TRPM4 | **2.342** | 9.80E-06 | **1.582** | 2.97E-02 |
| CLDN15 | **1.631** | 1.34E-04 | **1.582** | 3.94E-03 |
| KCNH8 | **3.742** | 6.63E-04 | **1.585** | 2.11E-02 |
| ARID4A | **2.395** | 4.15E-31 | **1.589** | 7.83E-07 |
| LRP5L | **2.175** | 1.33E-06 | **1.602** | 4.55E-02 |
| RRN3P3 | **1.646** | 1.81E-04 | **1.603** | 1.32E-02 |
| ADGRB2 | **2.350** | 4.95E-16 | **1.604** | 5.62E-04 |
| LINC00641 | **3.312** | 1.03E-41 | **1.604** | 2.61E-05 |
| FAM132B | **1.792** | 2.30E-07 | **1.606** | 1.32E-02 |
| DOCK6 | **3.289** | 2.69E-42 | **1.608** | 7.63E-06 |
| ZBTB20 | **4.019** | 2.75E-23 | **1.609** | 9.49E-05 |
| PDZD4 | **3.458** | 6.62E-40 | **1.609** | 3.42E-06 |
| KMT2B | **2.848** | 1.51E-24 | **1.615** | 5.67E-05 |
| HELLPAR | **10.781** | 2.39E-181 | **1.619** | 2.50E-06 |
| TBC1D32 | **1.932** | 2.81E-12 | **1.621** | 4.51E-04 |
| PSMD6-AS2 | **7.566** | 5.07E-29 | **1.622** | 1.27E-02 |
| JUNB | **9.090** | 2.71E-58 | **1.623** | 6.72E-05 |
| CSMD1 | **1.930** | 3.75E-69 | **1.623** | 7.38E-05 |
| TLE3 | **1.714** | 8.14E-12 | **1.626** | 1.95E-08 |
| SRCAP | **3.862** | 2.22E-11 | **1.634** | 2.08E-02 |
| XXyac-YR38GF2.1 | **2.546** | 5.73E-05 | **1.636** | 2.45E-02 |
| ATP6AP1L | **2.167** | 4.76E-12 | **1.638** | 4.85E-03 |
| SCAF4 | **1.710** | 1.04E-10 | **1.638** | 2.42E-06 |
| PCSK1N | **19.612** | 1.68E-35 | **1.639** | 3.48E-02 |
| SERTAD1 | **2.352** | 9.13E-10 | **1.643** | 2.17E-02 |
| LACC1 | **1.591** | 2.57E-05 | **1.644** | 1.51E-03 |
| PABPC1L | **3.927** | 1.46E-47 | **1.645** | 8.14E-08 |
| APLP1 | **3.721** | 4.21E-25 | **1.650** | 1.32E-04 |
| ABTB1 | **2.032** | 4.28E-04 | **1.652** | 3.23E-02 |
| ZNF703 | **4.168** | 9.05E-41 | **1.665** | 4.73E-02 |
| CDKN2B | **2.525** | 4.85E-21 | **1.670** | 2.92E-05 |
| MT-TP | **1.920** | 1.60E-05 | **1.682** | 2.88E-02 |
| ADAT2 | **3.011** | 2.41E-26 | **1.686** | 5.67E-05 |
| GPR153 | **4.169** | 1.26E-74 | **1.690** | 3.66E-07 |
| NAV2 | **3.416** | 3.69E-23 | **1.713** | 1.90E-15 |
| GDF15 | **7.903** | 8.47E-261 | **1.715** | 5.76E-03 |
| POLR2A | **3.678** | 5.87E-10 | **1.720** | 6.24E-03 |
| PLK3 | **2.017** | 1.23E-06 | **1.734** | 2.90E-04 |
| LTBP2 | **3.949** | 7.18E-10 | **1.738** | 1.26E-03 |
| SSH1 | **1.516** | 2.07E-14 | **1.743** | 7.20E-20 |
| DFNB31 | **4.678** | 8.34E-30 | **1.747** | 7.22E-04 |
| HSPE1P11 | **5.170** | 1.99E-05 | **1.747** | 1.29E-02 |
| LINC01318 | **10.232** | 7.54E-47 | **1.771** | 2.55E-03 |
| RP11-380B4.3 | **5.466** | 1.25E-16 | **1.773** | 2.46E-02 |
| KLRAP1 | **2.863** | 2.15E-10 | **1.776** | 1.17E-02 |
| AP000347.2 | **2.210** | 3.81E-05 | **1.777** | 2.48E-02 |
| AC000123.2 | **3.401** | 3.76E-26 | **1.782** | 2.22E-08 |
| GAD1 | **2.535** | 3.56E-20 | **1.812** | 4.63E-05 |
| DNHD1 | **2.725** | 4.66E-40 | **1.814** | 1.09E-10 |
| ONECUT1 | **2.037** | 1.25E-05 | **1.823** | 1.03E-03 |
| RP11-575G13.2 | **3.951** | 5.71E-19 | **1.842** | 4.50E-04 |
| TERT | **3.580** | 2.52E-09 | **1.849** | 3.55E-02 |
| HSP90AB4P | **1.877** | 5.13E-03 | **1.849** | 4.13E-02 |
| RP11-734K2.4 | **2.341** | 2.47E-04 | **1.850** | 2.38E-02 |
| RP11-310J24.3 | **9.733** | 1.04E-19 | **1.853** | 3.77E-02 |
| SHANK3 | **2.702** | 1.08E-19 | **1.854** | 1.16E-07 |
| SGK223 | **2.619** | 2.81E-09 | **1.859** | 4.42E-03 |
| AC005154.7 | **2.819** | 3.64E-03 | **1.863** | 3.35E-02 |
| PALM | **1.669** | 6.35E-03 | **1.868** | 1.46E-02 |
| GNG12-AS1 | **3.380** | 4.34E-10 | **1.870** | 3.77E-03 |
| CTB-152G17.6 | **7.173** | 1.33E-14 | **1.873** | 1.15E-02 |
| ZDHHC23 | **4.656** | 1.38E-37 | **1.875** | 7.70E-06 |
| TBX19 | **1.927** | 2.00E-06 | **1.880** | 4.21E-04 |
| RP11-547D13.1 | **2.420** | 6.61E-06 | **1.884** | 9.53E-04 |
| TRAPPC6A | **2.940** | 2.63E-10 | **1.888** | 1.08E-03 |
| MAT1A | **2.316** | 2.44E-07 | **1.890** | 1.88E-02 |
| RP11-390P2.2 | **15.281** | 3.35E-25 | **1.896** | 1.94E-02 |
| DGKH | **1.583** | 6.05E-21 | **1.899** | 3.30E-19 |
| RP11-70L8.5 | **16.365** | 1.70E-17 | **1.900** | 2.28E-02 |
| WNT2B | **3.049** | 7.62E-07 | **1.903** | 3.55E-02 |
| PTPRG | **1.546** | 1.18E-18 | **1.910** | 2.01E-23 |
| SLC9A5 | **2.072** | 2.11E-05 | **1.912** | 2.22E-03 |
| RP6-99M1.3 | **2.462** | 1.83E-03 | **1.913** | 3.02E-02 |
| ANKHD1-EIF4EBP3 | **3.779** | 3.17E-08 | **1.914** | 4.16E-02 |
| PARD6G-AS1 | **4.504** | 7.64E-09 | **1.918** | 1.83E-02 |
| COL7A1 | **3.071** | 1.76E-06 | **1.922** | 1.05E-03 |
| NPDC1 | **7.302** | 1.32E-33 | **1.923** | 2.39E-04 |
| LINC00174 | **3.841** | 5.98E-09 | **1.924** | 8.46E-03 |
| FZD7 | **1.574** | 2.38E-13 | **1.928** | 4.44E-13 |
| LINC01320 | **3.333** | 1.19E-12 | **1.929** | 2.27E-03 |
| NABP1 | **3.138** | 1.92E-84 | **1.931** | 3.35E-18 |
| AC007256.5 | **5.531** | 3.38E-22 | **1.932** | 1.71E-03 |
| CTC-425O23.5 | **2.197** | 8.00E-04 | **1.932** | 3.51E-02 |
| KCNC4 | **2.809** | 3.63E-22 | **1.941** | 2.20E-07 |
| MT-ND5 | **1.834** | 2.61E-04 | **1.973** | 1.33E-02 |
| ATHL1 | **3.765** | 6.82E-18 | **1.985** | 1.64E-07 |
| RP11-294J22.7 | **1.696** | 4.06E-03 | **1.986** | 9.38E-03 |
| SPRY4-IT1 | **1.582** | 1.94E-02 | **2.009** | 1.03E-02 |
| LURAP1L-AS1 | **2.784** | 9.14E-08 | **2.013** | 5.90E-04 |
| RP11-274B21.2 | **3.769** | 1.06E-11 | **2.020** | 1.19E-04 |
| SLC4A3 | **10.085** | 1.48E-45 | **2.056** | 1.61E-06 |
| CTA-414D7.1 | **3.138** | 1.31E-05 | **2.060** | 1.05E-02 |
| NAALADL2 | **3.377** | 3.18E-06 | **2.076** | 2.62E-05 |
| PLCB4 | **2.471** | 1.73E-21 | **2.078** | 7.35E-08 |
| RP11-6N13.1 | **4.173** | 1.44E-23 | **2.081** | 2.26E-03 |
| LINC00997 | **1.921** | 2.35E-02 | **2.083** | 5.55E-03 |
| MEGF6 | **53.052** | 1.05E-31 | **2.083** | 2.65E-02 |
| EGFL7 | **13.575** | 2.65E-73 | **2.089** | 2.39E-11 |
| KDM6B | **5.741** | 8.06E-15 | **2.092** | 1.25E-04 |
| B3GAT1 | **1.507** | 5.10E-03 | **2.098** | 9.99E-05 |
| PLEKHG5 | **1.773** | 2.19E-02 | **2.102** | 4.48E-03 |
| NIPAL1 | **3.234** | 1.36E-04 | **2.112** | 4.96E-03 |
| GPC1 | **8.451** | 9.15E-129 | **2.113** | 4.64E-18 |
| ENTPD1 | **4.836** | 2.60E-69 | **2.154** | 3.16E-02 |
| CCDC17 | **5.094** | 1.50E-12 | **2.156** | 2.62E-03 |
| MT-TV | **4.183** | 1.75E-07 | **2.157** | 2.75E-02 |
| AP3B2 | **1.930** | 1.70E-02 | **2.162** | 3.85E-02 |
| RP11-458F8.4 | **12.530** | 4.08E-10 | **2.174** | 4.10E-02 |
| GPR75 | **2.345** | 2.67E-04 | **2.182** | 2.43E-02 |
| SND1-IT1 | **4.029** | 4.19E-08 | **2.199** | 7.00E-04 |
| COL6A1 | **2.284** | 9.58E-16 | **2.214** | 1.27E-16 |
| RP4-800G7.2 | **2.254** | 9.68E-08 | **2.220** | 1.97E-07 |
| ADAM11 | **5.182** | 5.00E-23 | **2.234** | 1.49E-06 |
| EML6 | **1.935** | 5.46E-03 | **2.236** | 1.15E-02 |
| ZFP36 | **2.213** | 7.44E-06 | **2.239** | 3.97E-04 |
| C15orf52 | **4.454** | 1.59E-22 | **2.250** | 7.48E-05 |
| RPS3AP34 | **4.476** | 1.26E-08 | **2.255** | 4.78E-02 |
| RP11-454L9.2 | **2.083** | 2.34E-02 | **2.267** | 4.09E-02 |
| NUP210 | **2.001** | 3.21E-14 | **2.269** | 1.41E-05 |
| LINC00607 | **4.074** | 1.19E-09 | **2.285** | 1.49E-03 |
| RP11-164J13.1 | **3.677** | 5.80E-03 | **2.296** | 1.09E-02 |
| CTD-2587M2.1 | **5.266** | 6.71E-08 | **2.303** | 2.88E-02 |
| MYO15B | **3.025** | 4.69E-06 | **2.305** | 8.67E-06 |
| RP11-361A21.3 | **21.110** | 2.42E-18 | **2.306** | 2.70E-02 |
| C3orf35 | **2.451** | 2.75E-04 | **2.329** | 4.05E-03 |
| PLCL1 | **1.721** | 2.59E-03 | **2.337** | 3.20E-03 |
| RAB3IL1 | **1.795** | 9.25E-04 | **2.347** | 1.97E-03 |
| TRPM3 | **2.225** | 1.19E-05 | **2.363** | 4.85E-07 |
| PHLDB2 | **1.644** | 2.02E-18 | **2.395** | 5.57E-26 |
| LONRF3 | **1.555** | 2.74E-06 | **2.404** | 1.56E-02 |
| SEMA3D | **1.810** | 3.64E-03 | **2.434** | 3.83E-04 |
| LINC00342 | **6.434** | 2.85E-09 | **2.443** | 9.91E-03 |
| RP11-43D4.3 | **7.199** | 1.35E-13 | **2.446** | 1.01E-02 |
| HES1 | **1.587** | 1.49E-14 | **2.447** | 3.77E-33 |
| ZIC2 | **2.653** | 8.52E-05 | **2.471** | 5.35E-04 |
| DGKI | **1.848** | 5.17E-03 | **2.513** | 7.55E-03 |
| KCNC3 | **4.606** | 3.28E-04 | **2.517** | 2.04E-02 |
| ICAM5 | **9.117** | 8.79E-42 | **2.529** | 1.67E-06 |
| F12 | **6.239** | 9.35E-12 | **2.544** | 3.55E-03 |
| MYO5C | **4.239** | 9.45E-05 | **2.571** | 1.79E-02 |
| MEIS3P2 | **1.961** | 3.95E-03 | **2.580** | 4.53E-02 |
| VPS26BP1 | **5.032** | 3.64E-05 | **2.607** | 3.78E-02 |
| PRRX1 | **2.230** | 3.11E-23 | **2.611** | 5.95E-14 |
| TFAMP2 | **13.092** | 2.36E-12 | **2.619** | 1.98E-02 |
| ADAMTS7 | **1.998** | 3.22E-04 | **2.621** | 3.62E-05 |
| DNAH1 | **1.618** | 1.47E-04 | **2.623** | 2.00E-10 |
| VASH1 | **1.961** | 8.01E-13 | **2.639** | 3.80E-19 |
| RP11-274B21.10 | **4.877** | 1.43E-03 | **2.657** | 3.27E-02 |
| RP11-117N2.2 | **2.699** | 1.47E-03 | **2.688** | 4.71E-02 |
| FSIP2-AS1 | **3.550** | 2.05E-02 | **2.693** | 4.45E-02 |
| KCNJ14 | **1.767** | 1.97E-02 | **2.769** | 3.89E-04 |
| AC064875.2 | **3.746** | 9.67E-13 | **2.775** | 2.51E-02 |
| PAQR6 | **2.273** | 1.34E-03 | **2.821** | 4.85E-03 |
| HSPE1P26 | **3.775** | 5.21E-05 | **2.826** | 2.03E-02 |
| AP001205.1 | **4.263** | 1.83E-04 | **2.835** | 2.92E-02 |
| TMEM145 | **12.965** | 3.57E-15 | **2.921** | 1.39E-04 |
| MRC2 | **1.580** | 1.82E-02 | **2.925** | 3.13E-16 |
| SEPT14P12 | **1.980** | 1.77E-02 | **2.937** | 1.78E-02 |
| TOX3 | **3.007** | 2.85E-03 | **2.984** | 4.87E-04 |
| SMARCD3 | **1.800** | 8.35E-08 | **3.007** | 3.70E-12 |
| ADM5 | **6.899** | 1.08E-04 | **3.030** | 3.61E-02 |
| BCAM | **3.861** | 1.15E-05 | **3.030** | 1.06E-04 |
| PHF21B | **2.751** | 1.13E-06 | **3.033** | 1.38E-03 |
| APC2 | **7.259** | 2.23E-05 | **3.043** | 4.30E-03 |
| LL22NC03-2H8.5 | **15.815** | 1.51E-15 | **3.081** | 4.48E-04 |
| TMEM151A | **4.883** | 1.26E-03 | **3.085** | 1.38E-02 |
| ACTN2 | **11.058** | 6.03E-19 | **3.107** | 1.07E-02 |
| B4GALNT4 | **3.390** | 4.51E-04 | **3.120** | 1.62E-02 |
| FLJ46284 | **5.755** | 9.42E-17 | **3.121** | 6.63E-11 |
| CEP126 | **4.371** | 1.16E-04 | **3.184** | 6.46E-03 |
| RP11-386B1.1 | **9.274** | 2.36E-09 | **3.193** | 1.17E-02 |
| TEX15 | **2.926** | 4.47E-31 | **3.202** | 4.07E-02 |
| RP11-59E19.4 | **2.728** | 4.98E-03 | **3.252** | 4.81E-02 |
| KANK3 | **4.209** | 4.86E-03 | **3.265** | 4.04E-02 |
| DSP | **2.023** | 7.44E-04 | **3.315** | 4.81E-03 |
| STEAP2 | **2.035** | 1.49E-09 | **3.345** | 1.91E-03 |
| SATB1 | **2.329** | 3.44E-05 | **3.373** | 2.11E-02 |
| CHRM3 | **4.367** | 9.42E-03 | **3.408** | 2.39E-02 |
| OR4F15 | **7.436** | 1.15E-04 | **3.423** | 1.89E-02 |
| TOR4A | **3.006** | 7.17E-03 | **3.459** | 4.84E-02 |
| WNT5A | **2.383** | 1.10E-06 | **3.480** | 2.80E-02 |
| TXK | **2.652** | 2.85E-02 | **3.481** | 1.52E-03 |
| EGR2 | **9.036** | 2.44E-42 | **3.553** | 3.34E-09 |
| CCDC146 | **3.187** | 3.11E-09 | **3.556** | 3.93E-11 |
| EXPH5 | **1.612** | 2.36E-06 | **3.653** | 1.17E-23 |
| AL121989.1 | **4.270** | 3.88E-03 | **3.656** | 4.79E-02 |
| WNK2 | **3.607** | 1.20E-14 | **3.679** | 1.72E-07 |
| FERMT3 | **3.292** | 2.04E-02 | **3.685** | 3.16E-02 |
| PTGS2 | **2.969** | 1.97E-39 | **3.697** | 1.89E-31 |
| BEGAIN | **8.972** | 3.57E-31 | **3.711** | 4.51E-04 |
| ANK3 | **9.780** | 1.71E-06 | **3.741** | 1.43E-02 |
| JPH1 | **1.663** | 1.68E-03 | **3.744** | 2.43E-02 |
| OBSCN | **1.924** | 5.20E-06 | **3.783** | 2.96E-18 |
| SLC30A3 | **1.882** | 3.15E-02 | **3.793** | 3.68E-02 |
| COL6A2 | **6.001** | 1.05E-38 | **3.896** | 1.02E-07 |
| CKB | **2.344** | 1.01E-07 | **3.968** | 1.95E-07 |
| RP11-307P5.1 | **3.373** | 3.95E-03 | **3.975** | 2.92E-02 |
| BTG2 | **1.574** | 3.30E-04 | **4.032** | 2.33E-10 |
| ZSWIM5 | **3.437** | 4.93E-05 | **4.035** | 1.29E-02 |
| CPLX1 | **4.550** | 1.39E-02 | **4.053** | 2.54E-02 |
| CPNE7 | **3.944** | 2.71E-05 | **4.082** | 1.61E-04 |
| AC010970.2 | **8.690** | 3.65E-04 | **4.086** | 2.64E-02 |
| RP11-44N11.1 | **3.120** | 2.66E-02 | **4.220** | 3.39E-02 |
| REEP1 | **1.905** | 2.35E-02 | **4.259** | 1.38E-02 |
| SNED1 | **3.979** | 9.33E-07 | **4.356** | 1.84E-24 |
| APBA1 | **5.422** | 1.36E-04 | **4.368** | 8.71E-03 |
| POU3F3 | **9.867** | 6.66E-21 | **4.507** | 6.37E-03 |
| SPEG | **1.729** | 2.01E-02 | **4.613** | 1.64E-08 |
| C3orf80 | **3.833** | 6.04E-03 | **4.614** | 8.98E-03 |
| TLE2 | **6.730** | 1.91E-06 | **4.642** | 1.13E-04 |
| KCNQ5-IT1 | **7.389** | 5.99E-21 | **4.679** | 2.34E-04 |
| KDR | **4.269** | 1.60E-36 | **4.681** | 5.55E-20 |
| RP11-313J2.1 | **4.115** | 3.29E-21 | **4.710** | 9.02E-14 |
| RP11-54O7.17 | **3.575** | 1.15E-02 | **4.790** | 2.49E-03 |
| KIAA1456 | **2.444** | 4.92E-68 | **5.014** | 5.01E-30 |
| NXPH4 | **4.277** | 2.31E-06 | **5.064** | 2.99E-05 |
| C1QL1 | **16.177** | 2.54E-27 | **5.095** | 9.62E-13 |
| SRRM3 | **2.112** | 7.02E-03 | **5.116** | 9.14E-09 |
| PCSK1 | **3.343** | 5.10E-04 | **5.136** | 6.21E-07 |
| SLC4A11 | **3.867** | 4.70E-04 | **5.165** | 1.98E-03 |
| ARHGAP29 | **2.070** | 7.73E-26 | **5.223** | 1.34E-33 |
| RP11-120M18.2 | **4.066** | 4.66E-15 | **5.296** | 1.18E-22 |
| SEZ6L2 | **2.772** | 7.00E-03 | **5.387** | 3.91E-14 |
| LAMA5 | **19.238** | 2.82E-74 | **5.413** | 5.51E-32 |
| STON2 | **2.921** | 5.23E-05 | **5.419** | 4.39E-16 |
| SPTBN2 | **1.969** | 1.74E-12 | **5.493** | 2.10E-52 |
| THSD7A | **2.542** | 4.93E-78 | **5.669** | 2.80E-06 |
| FLRT2 | **1.835** | 2.00E-15 | **5.695** | 3.96E-22 |
| IL1RAPL1 | **3.051** | 1.49E-36 | **6.020** | 8.16E-57 |
| RORA | **7.249** | 1.07E-114 | **6.476** | 3.96E-10 |
| SALL4 | **5.311** | 1.82E-04 | **6.480** | 5.44E-08 |
| SYT7 | **5.939** | 1.24E-13 | **6.616** | 2.27E-06 |
| CASKIN1 | **3.343** | 1.71E-08 | **6.690** | 1.84E-09 |
| RP11-444D3.1 | **2.135** | 1.23E-09 | **6.921** | 8.75E-16 |
| RP11-369K16.1 | **5.294** | 4.04E-16 | **7.284** | 4.47E-05 |
| LINGO2 | **2.742** | 1.59E-07 | **7.446** | 3.88E-05 |
| CTD-3247F14.2 | **3.425** | 4.82E-03 | **7.965** | 1.11E-10 |
| GDNF-AS1 | **6.424** | 5.81E-04 | **9.334** | 2.72E-05 |
| CACNB4 | **8.052** | 7.29E-11 | **9.586** | 1.05E-07 |
| LPL | **1.712** | 1.16E-02 | **12.386** | 2.06E-84 |
| PKHD1 | **5.656** | 3.61E-72 | **39.738** | 7.97E-28 |
