# Supplementary material for: Loss of Spry1 reduces growth of BRAFV600-mutant cutaneous melanoma and improves response to targeted therapy
Source: Cell Death Dis. 2020 May 22;11(5):392. doi: 10.1038/s41419-020-2585-y (PMC7244546; doi:10.1038/s41419-020-2585-y)
Supplement: Supplementary file 4 — Supplementary Table 3 [file 41419_2020_2585_MOESM4_ESM.doc]

Supplementary Table 3. Antibodies used for Western Blot analyses.

| **Antibody name** | **Vendor** | **Catalogue number** |
| --- | --- | --- |
| Rabbit anti-phospho-Mek1/2 (Ser217/221) (41G9) | Cell Signaling Technology | #9154 |
| Rabbit anti-Mek1/2 (47E6) | Cell Signaling Technology | #9126 |
| Rabbit anti-phospho-p42/44 MAPK (Erk1/2) (Thr202/Tyr204) | Cell Signaling Technology | #9101 |
| Rabbit anti-p42/44 MAPK (Erk1/2) | Cell Signaling Technology | #9102 |
| Rabbit anti-phospho-p38 MAPK (Thr180/Tyr182) | Cell Signaling Technology | #9211 |
| Rabbit anti-p38 MAPK (D13E1) | Cell Signaling Technology | #8690 |
| Rabbit anti-Spry1 (D9V6P) | Cell Signaling Technology | #13013 |
| Rabbit anti-non-phospho (Active) β-Catenin (Ser33/37/Thr41) (D13A1) | Cell Signaling Technology | #8814 |
| Rabbit anti-β-Catenin (D10A8) | Cell Signaling Technology | #8480 |
| Rabbit anti-p53 | Cell Signaling Technology | #9282 |
| Rabbit anti-MMP2 (D8N9Y) | Cell Signaling Technology | #13132 |
| Mouse anti-Bcl2 (124) | Cell Signaling Technology | #15071 |
| Mouse anti-β Tubulin (D3U1W) | Cell Signaling Technology | #86298 |
| Rabbit anti-Slug (C19G7) | Cell Signaling Technology | #9585 |
| Rabbit anti-Axl (C2B12) | Cell Signaling Technology | #4939 |
| Mouse anti-γHistone H2AX (Ser139) | Santa Cruz Biotechnology | sc-517348 |
| Mouse anti-Cyclin D1 (DCS-6) | Santa Cruz Biotechnology | sc-20044 |
| Mouse anti-Twist (Twist2C1a) | Santa Cruz Biotechnology | sc-81417 |
