# Supplementary material for: Loss of Spry1 reduces growth of BRAFV600-mutant cutaneous melanoma and improves response to targeted therapy
Source: Cell Death Dis. 2020 May 22;11(5):392. doi: 10.1038/s41419-020-2585-y (PMC7244546; doi:10.1038/s41419-020-2585-y)
Supplement: Supplementary file 5 — Supplementary Table 4 [file 41419_2020_2585_MOESM5_ESM.doc]

Supplementary Table 4. Primer used for RT-PCR analyses

| Gene | Forward | Reverse |
| --- | --- | --- |
| AEBP1 | CGAAGAGAAGGAGGAGCTGA | CTTTCGGGGCTCTTTGTG |
| BTG2 | GCGAGCAGAGGCTTAAGGT | GGGAAACCAGTGGTGTTTGTA |
| DNAJA4 | CCCTGGAAAAAGGGATTCTG | TCCTCACTTTCTGTCGAGGAG |
| DNAJC15 | TGGTGTCATCGCTCCAGTTG | ATGCGTAGCGACCTGCAAAT |
| MMP-2 | CCCCAAAACGGACAAAGAG | CTTCAGCACAAACAGGTTGC |
| Spry1 | CCTCCTGAACTTTTAGCTTTCAA | GGAAAAATTCTAAAGAAAACAAAAACA |
| β-actin | CGAGCGCGGCTACAGCTT | CCTTAATGTCACGCACGATT |
