# Supplementary figures and images for: Loss of Spry1 reduces growth of BRAFV600-mutant cutaneous melanoma and improves response to targeted therapy
Source: Cell Death Dis. 2020 May 22;11(5):392. doi: 10.1038/s41419-020-2585-y (PMC7244546; doi:10.1038/s41419-020-2585-y)

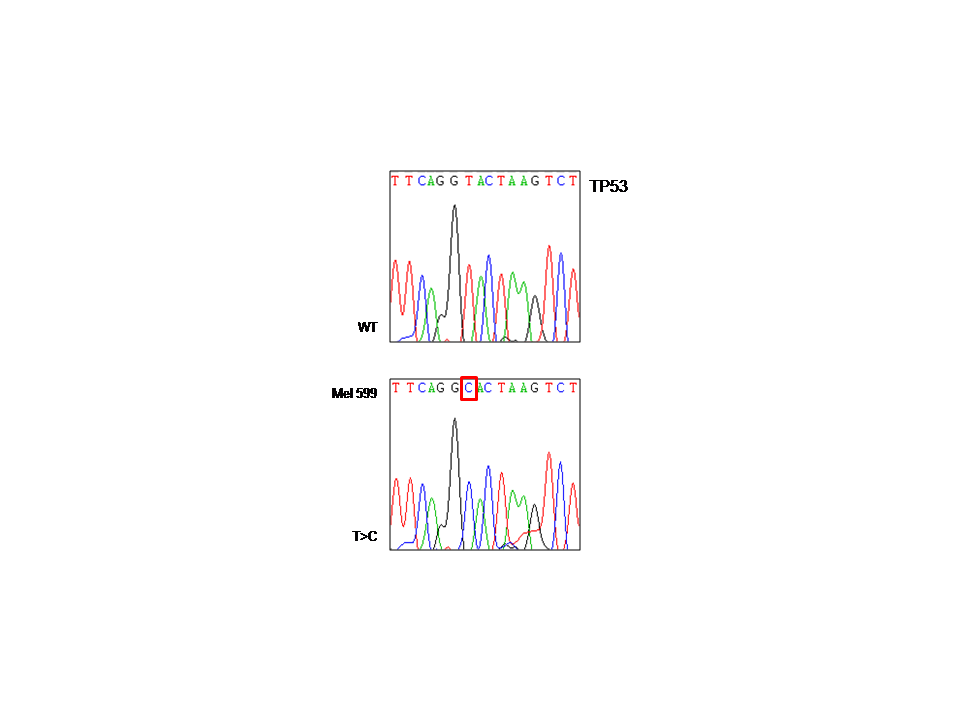

Supplement: Supplementary file 6 — Supplementary Figure S1 [file 41419_2020_2585_MOESM6_ESM.tif]

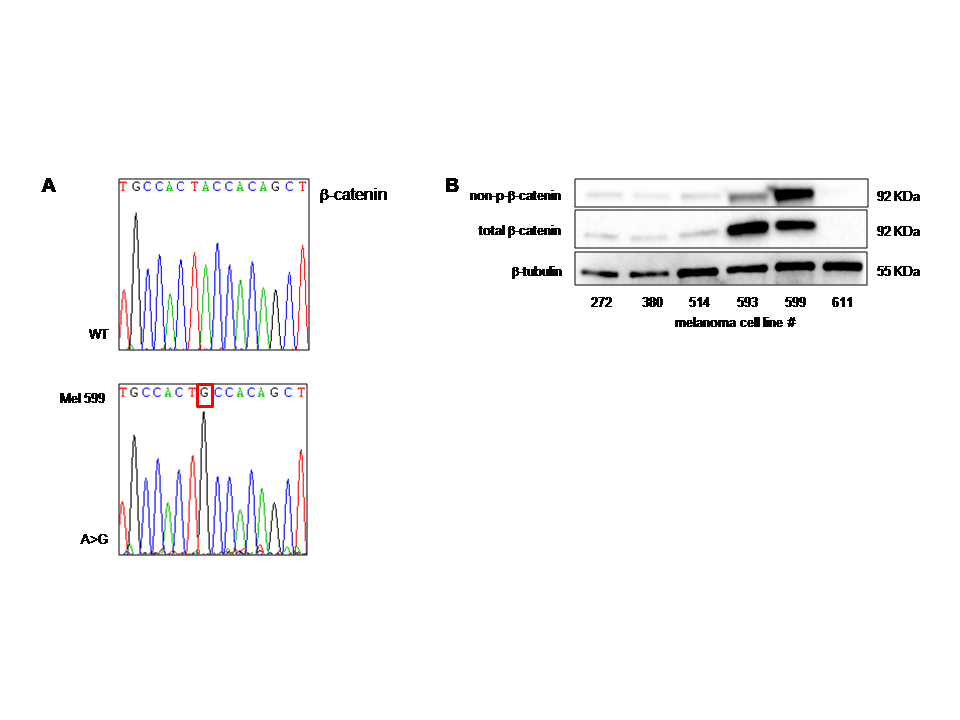

Supplement: Supplementary file 7 — Supplementary Figure S2 [file 41419_2020_2585_MOESM7_ESM.tif]

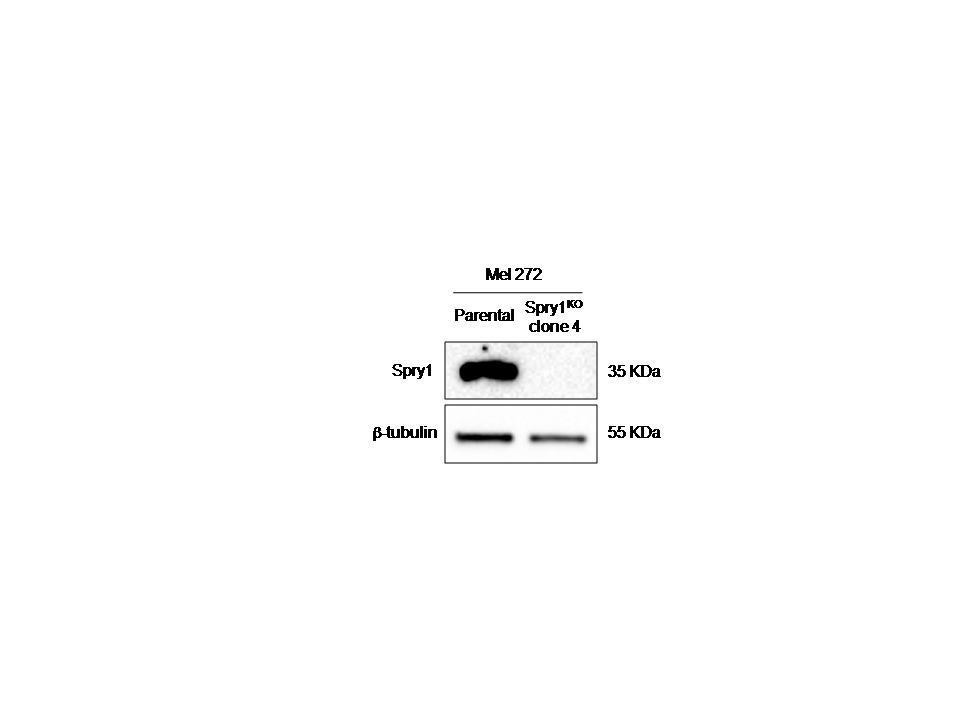

Supplement: Supplementary file 8 — Supplementary Figure S3 [file 41419_2020_2585_MOESM8_ESM.tif]

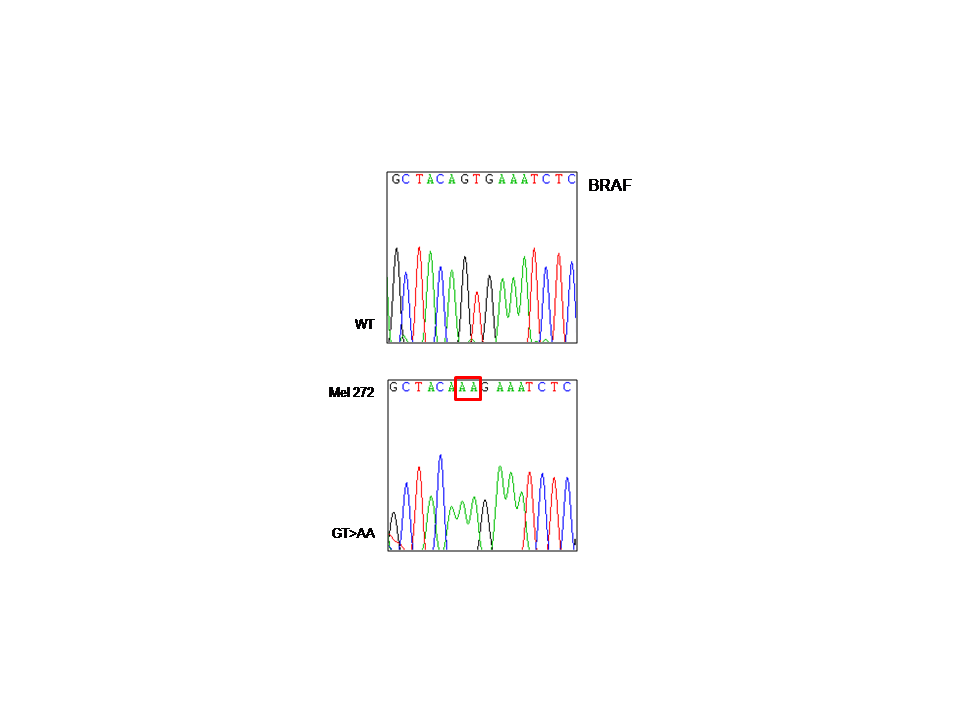

Supplement: Supplementary file 9 — Supplementary Figure S4 [file 41419_2020_2585_MOESM9_ESM.tif]

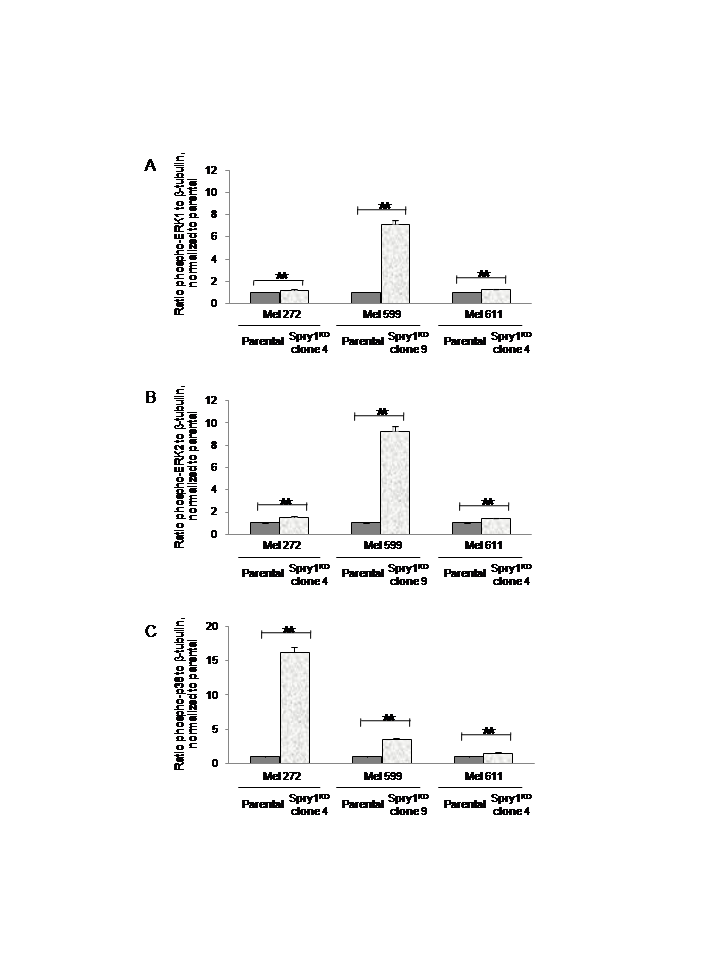

Supplement: Supplementary file 10 — Supplementary Figure S5 [file 41419_2020_2585_MOESM10_ESM.tif]

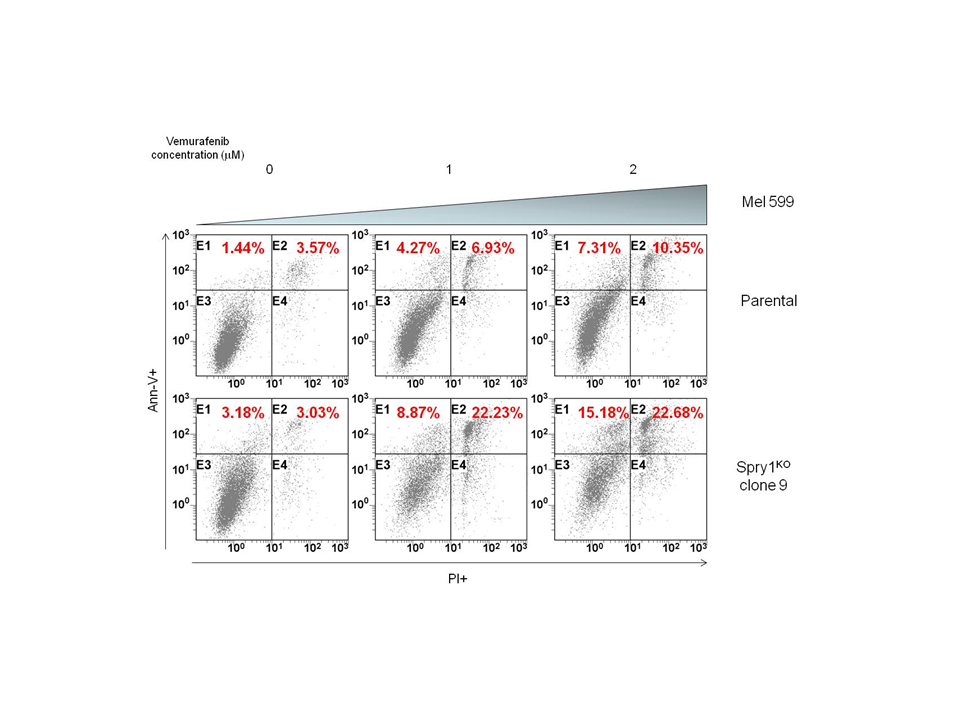

Supplement: Supplementary file 11 — Supplementary Figure S6 [file 41419_2020_2585_MOESM11_ESM.tif]

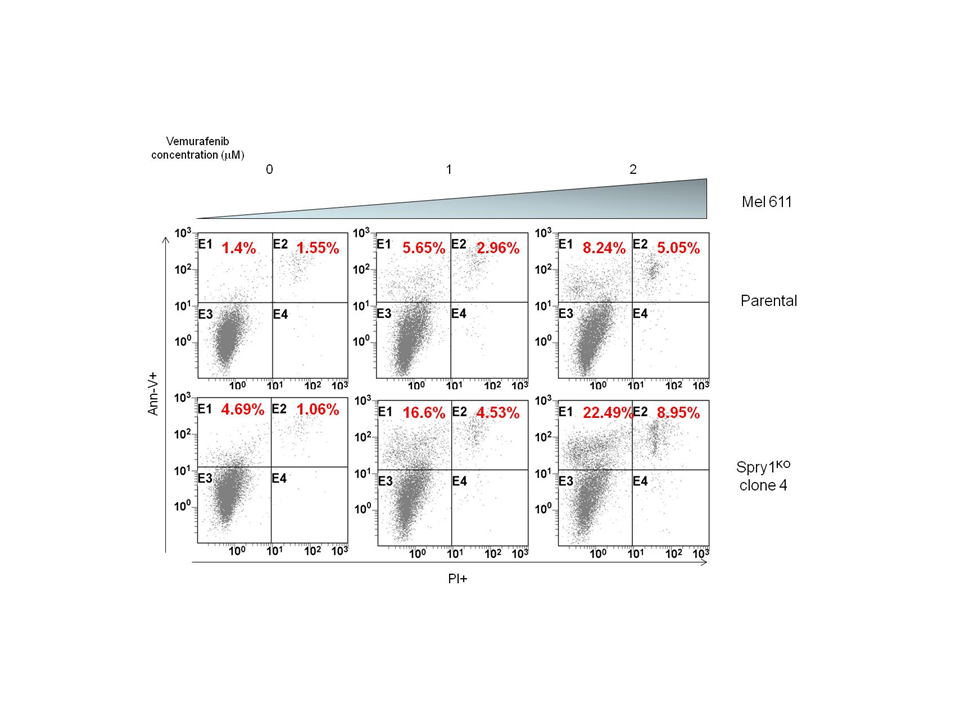

Supplement: Supplementary file 12 — Supplementary Figure S7 [file 41419_2020_2585_MOESM12_ESM.tif]

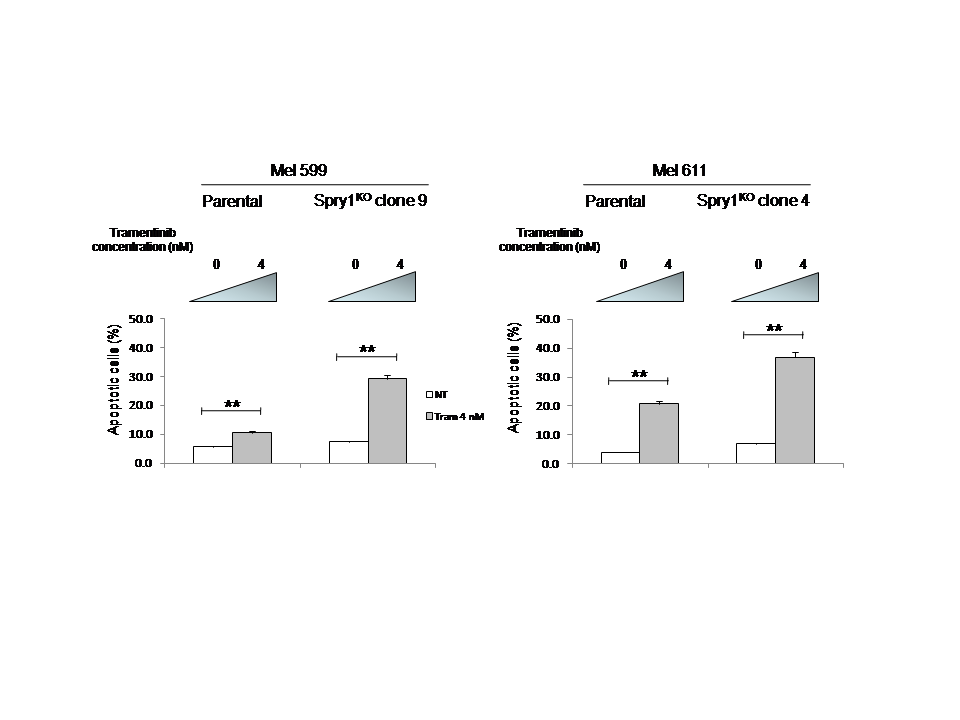

Supplement: Supplementary file 13 — Supplementary Figure S8 [file 41419_2020_2585_MOESM13_ESM.tif]

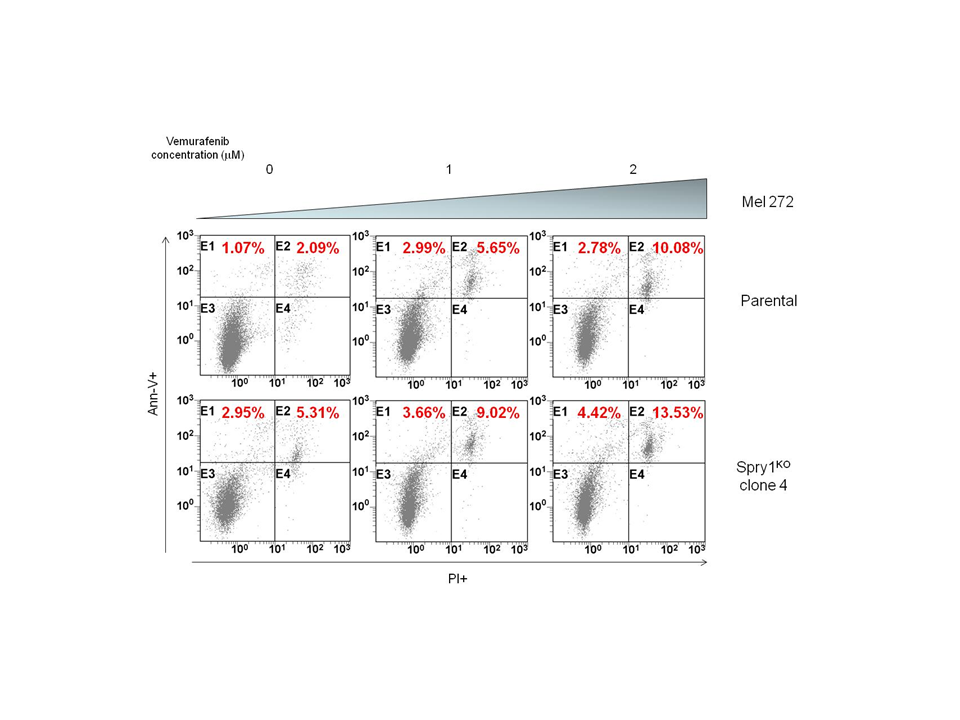

Supplement: Supplementary file 14 — Supplementary Figure S9 [file 41419_2020_2585_MOESM14_ESM.tif]

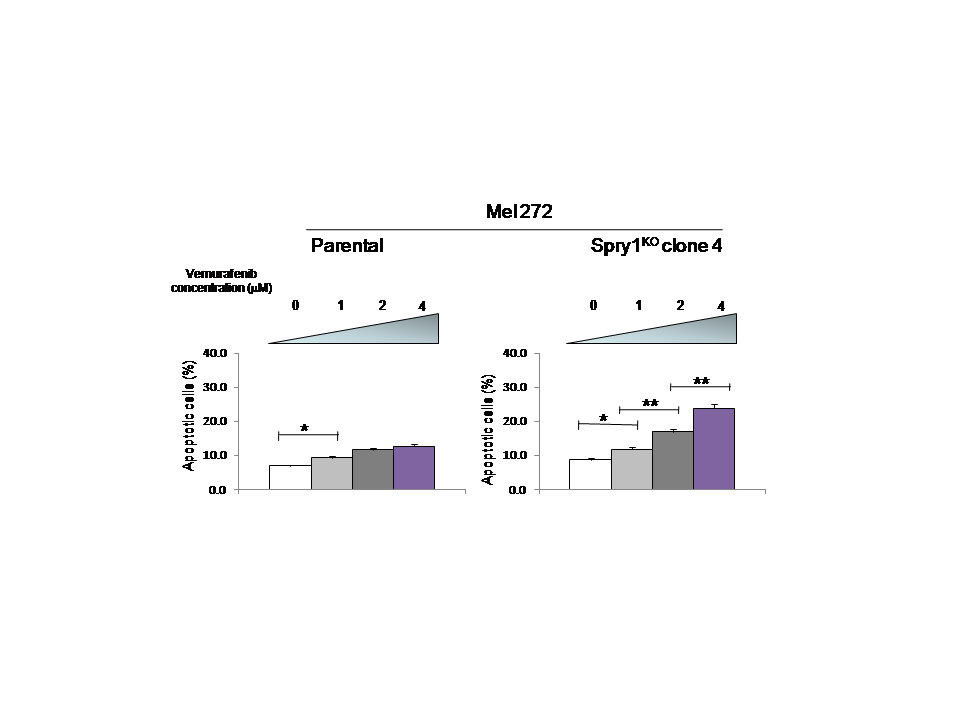

Supplement: Supplementary file 15 — Supplementary Figure S10 [file 41419_2020_2585_MOESM15_ESM.tif]

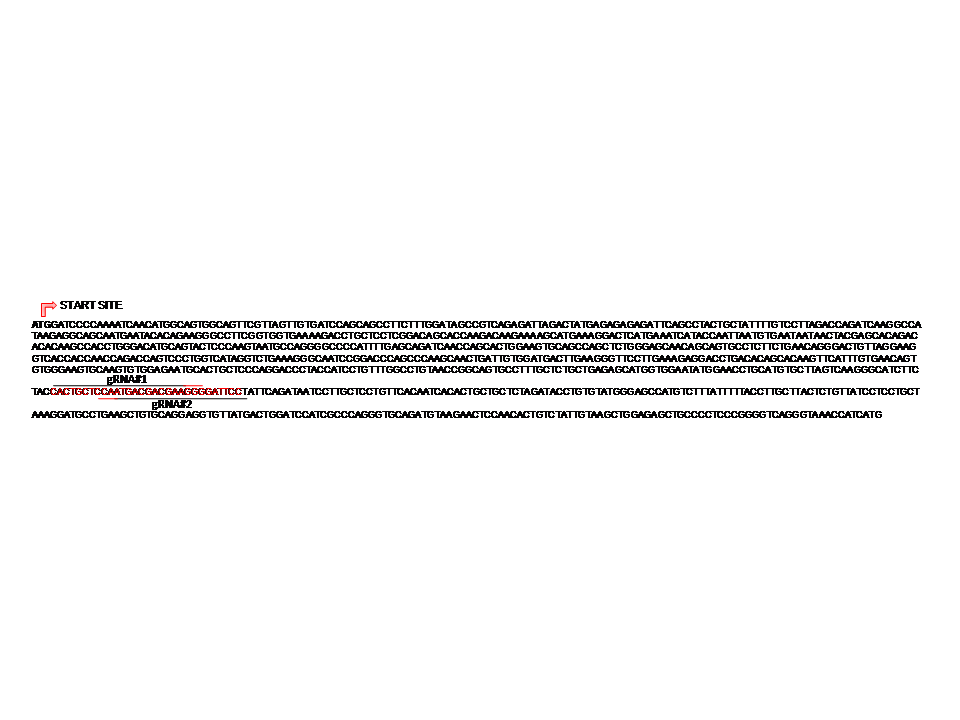

Supplement: Supplementary file 16 — Supplementary Figure S11 [file 41419_2020_2585_MOESM16_ESM.tif]
